# Zinc finger protein X‐linked promotes expansion of EpCAM^+^ cancer stem‐like cells in hepatocellular carcinoma

**DOI:** 10.1002/1878-0261.12036

**Published:** 2017-03-29

**Authors:** Chao Wang, Si‐yuan Fu, Ming‐da Wang, Wen‐bo Yu, Qin‐shu Cui, Hong‐ru Wang, Hai Huang, Wei Dong, Wei‐wei Zhang, Peng‐peng Li, Chuan Lin, Ze‐ya Pan, Yuan Yang, Meng‐chao Wu, Wei‐ping Zhou

**Affiliations:** ^1^ The Third Department of Hepatic Surgery Eastern Hepatobiliary Hospital Second Military Medical University Shanghai China; ^2^ Department of Urology Changhai Hospital Second Military Medical University Shanghai China; ^3^ The Department of Hepatic Surgery Eastern Hepatobiliary Hospital Second Military Medical University Shanghai China; ^4^ The Second Military Medical University Shanghai China; ^5^ Department of Urinary Surgery Changzheng Hospital Second Military Medical University Shanghai China; ^6^ Liberty Mutual Group Boston MA USA; ^7^ Department of Laboratory Diagnostic Changhai Hospital Second Military Medical University Shanghai China

**Keywords:** cancer stem‐like cells, EpCAM, hepatocellular carcinoma, Stemness, ZFX, β‐catenin

## Abstract

Zinc finger protein X‐linked (ZFX) is frequently upregulated in multiple human malignancies and also plays a critical role in the maintenance of self‐renewal in embryonic stem cells. However, the role of ZFX in liver cancer stem cells (CSCs) remains obscure. We observed that the elevated expression of both ZFX and epithelial cell adhesion molecule (EpCAM) was associated with aggressive clinicopathological features and indicated poor prognosis in patients with hepatocellular carcinoma (HCC). ZFX was commonly enriched in liver EpCAM^+^ CSCs. Knockdown of ZFX decreased the proportion of EpCAM^+^ CSCs in HCC cells and suppressed their expression of stemness‐related genes, self‐renewal capacity, chemoresistance, metastatic potential, and tumorigenicity. Conversely, upregulation of ZFX in CSCs rescued these inhibitory effects and enhanced stem‐like properties. Mechanistically, depletion of ZFX reduced nuclear translocation and transactivation of β‐catenin, thereby inhibiting the self‐renewal capacity of EpCAM^+^ CSCs. Moreover, knockdown of β‐catenin attenuated the self‐renewal of EpCAM^+^ HCC cells stably expressing ZFX, further indicating that β‐catenin is required for ZFX‐mediated expansion and maintenance of EpCAM^+^ CSCs. Taken together, our findings indicate that ZFX activates and maintains EpCAM^+^ liver CSCs by promoting nuclear translocation and transactivation of β‐catenin. Furthermore, combination of ZFX and EpCAM may serve as a significant indicator for prognosis of patients with HCC.

**Abbreviations**
APC: adenomatous polyposis coli
CSCs: cancer stem‐like cells
DDP: cisplatin
DFS: disease‐free survival
ESCs: pluripotent embryonic stem cells
GSK‐3β: glycogen synthase kinase 3β
HCC: hepatocellular carcinoma
HSCs: hematopoietic stem cells
LV: lentivirus
OS: overall survival
RT‐PCR: real‐time polymerase chain reaction
TMA: tissue microarray
ZFX: zinc finger X‐chromosomal protein

## 1 Introduction

Hepatocellular carcinoma (HCC) ranks as the sixth most common cancer and is the third leading cause of cancer‐related death worldwide (Jemal *et al*., 2011). Although surgical resection and liver transplantation are curative treatments for patients with HCC (Carr, 2004; Kassahun *et al*., 2006), most HCCs are often diagnosed at a late stage when these therapies are no longer effective options. For the patients with nonresectable HCCs, chemotherapy via either transarterial chemoembolization or a systemic route is the most commonly given treatment. However, the overall response and long‐term survival rate of HCC remains unsatisfactory due to the rapid progression and highly acquired chemoresistant nature (Aravalli *et al*., 2008). Accumulating evidence has suggested that the presence of cancer stem‐like cells (CSCs) is responsible for chemoresistance and relapse of HCC (Chiba *et al*., 2009; Ma *et al*., 2010; Oishi and Wang, 2011; Tong *et al*., 2011). The theory of CSCs proposes that tumors are maintained in a hierarchical organization of cells, in which a subset of CSCs are indispensable to initiate and sustain tumor growth because of their strong self‐renewal capacity (Jordan *et al*., 2006). The existence of CSCs was first observed in myeloid leukemia and more recently in a variety of solid malignancies, including HCCs (Lee *et al*., 2011; Visvader, 2011). Several lines of evidence indicate the significance of liver CSCs in hepatocarcinogenesis (Lee *et al*., 2011; Marquardt and Thorgeirsson, 2010; Yang *et al*., 2008). It is widely acknowledged that a variety of cell surface antigens such as EpCAM (Yamashita *et al*., 2009), CD133 (Ma *et al*., 2007), CD90 (Yang *et al*., 2008), CD24 (Lee *et al*., 2011), and OV6 (Yang *et al*., 2008) could be used for identification and characterization of CSCs. Thus, targeting liver CSC to sensitize them to chemotherapy may be exploited for novel therapeutic paradigms for patients with HCCs.

Zinc finger X‐chromosomal protein (ZFX) is a zinc finger transcription factor encoded on mammalian X chromosome and is highly conserved among vertebrates. It contains an acidic transcriptional activation domain (AD), a nuclear localization sequence (NLS), and a DNA binding domain consisting of 13 C2H2‐type zinc fingers at the carboxyl terminus (Schneider‐Gadicke *et al*., 1989). A growing body of evidence shows that ZFX is frequently upregulated in a variety of human malignancies and is associated with poor prognosis (Jiang and Liu, 2015; Li *et al*., 2015a,b; Weng *et al*., 2015). Meanwhile, other investigations in different cancer cell lines also revealed that knockdown of ZFX apparently induced cell cycle arrest and inhibited cell proliferation and migration (Fang *et al*., 2012; Li *et al*., 2013; Wu *et al*., 2013). In addition to the elevated levels of ZFX in human cancers, overexpression of ZFX is also observed in pluripotent embryonic stem cells (ESCs) and hematopoietic stem cells (HSCs). Previous studies have demonstrated that ZFX maintains the self‐renewal potential of ESCs and HSCs via blocking cell differentiation (Galan‐Caridad *et al*., 2007; Young, 2011). Moreover, a genomewide siRNA screen identifies an alternative module in self‐renewal transcription network formed by ZFX, c‐Myc, and other cofactors in mouse ESCs (Hu *et al*., 2009). More importantly, a recent study has demonstrated the contribution of ZFX in the maintenance of stem‐like features of HCC cells (Lai *et al*., 2014). Mechanistically, ZFX binds on the promoters of two important transcriptional factors, Nanog and Sox2, thus enforcing their expressions in HCCs. However, the expression and role of ZFX in liver CSCs have yet to be elucidated.

In this study, we investigated the functional role of ZFX in maintaining stem‐like properties of liver putative CSCs during HCC progression. Here, we reported that ZFX is preferentially expressed in liver CSCs and correlated with poor prognosis in patients with HCC. Meanwhile, knockdown of ZFX contributes to the reduction of liver CSCs by suppressing the self‐renewal capacity, drug resistance property, metastatic potential, and tumorigenicity *in vivo*. Furthermore, we determined how ZFX confers these stemness characteristics in liver CSCs. Our data indicate that ZFX may play a crucial role in maintaining stem‐like properties of EpCAM^+^ CSCs in HCC through facilitating nuclear translocation and transactivation of β‐catenin. Therefore, our findings provide a novel insight for the relationship between ZFX and stem‐like characteristics of CSCs during hepatocarcinogenesis. Meanwhile, inhibition of ZFX may be exploited to suppress HCC progression by inactivating CSC subpopulations.

## 2 Materials and Methods

For details of Materials and Methods, see the information of the Supporting information.

### 2.1 HCC specimens and tissue microassays

All patient samples were obtained following informed consent according to an established protocol approved by the Ethic Committee of Eastern Hepatobiliary Surgery Hospital (Shanghai, China). The HCC tissue microassays containing 242 paired HCC tissues were constructed as described previously (Wen *et al*., 2012). Overall survival (OS) was the interval between the dates of surgery and death. Disease‐free survival (DFS) was the interval between the dates of surgery and recurrence. In addition, another 50 and 18 paired HCC samples were collected for RT‐PCR and western blotting, respectively. Human primary HCC cells were dissociated from fresh HCC specimens as described previously (Wang *et al*., 2015).

### 2.2 Flow cytometry and magnetic cell sorting

The magnetic‐activated cell sorting and flow cytometric assay were performed with a MoFlo XDP Cell Sorter (Beckman, Boulevard Brea, CA, USA). HCC cell lines or primary HCC cells were magnetically labeled with APC‐conjugated EpCAM antibody (mouse IgG1; Miltenyi Biotec, CA, USA). Then, cells were subsequently incubated with rat anti‐mouse IgG1 MicroBeads and separated on MACS MS column (Miltenyi Biotec, Teterow, Germany). All the procedures were carried out according to the manufacturer's instructions. HCC cells infected with LV‐shCON or LV‐shZFX were incubated with EpCAM antibody, and percentages of EpCAM‐positive cells were detected by flow cytometry. The apoptotic rates of HCC cells treated with DDP (1 μg·mL^−1^) for 48 h were quantified using flow cytometry as previously reported (Qian *et al*., 2012).

### 2.3 *In* *vivo* xenograft assay and pulmonary metastasis model

Six‐week‐old male nude mice and severe combined immunodeficient NOD/SCID mice were purchased from Chinese Science Academy (Shanghai, China). All animal experiments were performed according to the Second Military Medical University Animal Care Facility and the National Institutes of Health guidelines. EpCAM^+^ Huh7 shZFX and control cells were subcutaneously injected into right flank of nude mice at different cell numbers as indicated. The incidence and volumes of subcutaneous tumors were monitored every four days. All the mice were killed at 6 weeks postinoculation for the assessment of tumor incidence and size. Formula: *V* = (width)^2 ^× length/2 (mm^3^). For HCC‐derived bioluminescent assay, indicated HCC cells were labeled with luciferase and imaging was performed with a Xenogen IVIS 100 cooled CCD camera (PerkinElmer Inc., Hopkinton, MA, USA) on Day 44. The mice were given intraperitoneal injections with 200 μL of 15 mg·mL^−1^ D‐luciferin 15 min before imaging, and then, the mice were placed in a lighttight chamber, the acquisition time ranged from 3 s to 1 min, and pseudoimages of the emitted light in photons·s^−1^cm^−2^ per steradian superimposed over the grayscale photographs of the animal were taken. For *in vivo* metastasis assay, the indicated HCC cells were injected into the tail vein of mice for the establishment of pulmonary metastatic model. Mice were killed at 8 weeks postinjection, and the lung metastatic foci were detected microscopically by H&E staining.

### 2.4 Statistical analysis

Differences between variables were assessed by two‐tailed Student's *t*‐test or ANOVA. The patient survival of distinct subgroups was calculated by the Kaplan–Meier method and log‐rank analysis. All the values were expressed as mean ± SD, and statistical significance was defined as *P* < 0.05. Data analysis was performed by the spss software (version 16; SPSS, Armonk, NY, USA).

## 3 Results

### 3.1 ZFX is commonly upregulated in HCC tissues and serves as a significant indicator of poor prognosis in patients with HCC

**Figure 1:**
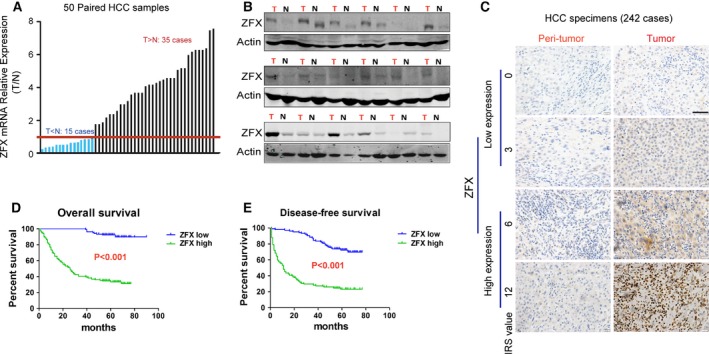
ZFX is frequently upregulated in HCC tissues and predicts poor prognosis in patients with HCC. (A) The expression levels of ZFX mRNA in 50 paired HCC and their adjacent nontumorous tissues were evaluated by qRT‐PCR. (B) The expression of ZFX protein in tumor samples (T) and their corresponding peri‐tumoral tissues (N) from 18 patients with HCC were analyzed by western blots. (C) Representative IHC staining of ZFX expression in peri‐tumoral tissue and HCC samples is shown (scale bar = 50 μm). (D,E) Kaplan–Meier analysis of the association between ZFX expression levels and disease‐free survival (DFS) or overall survival (OS) in patients with HCCs (*n* = 242).

To verify the underlying role of ZFX in liver tumorigenesis, we first determined the expression levels of ZFX in 50 pairs of HCC specimens and adjacent nontumorous liver tissues via RT‐PCR. We observed that ZFX was commonly overexpressed in HCC tissues compared with that in their matched nontumorous liver sections (Fig. 1A). Western blot analysis also confirmed the elevated protein levels of ZFX in most HCC cases (Fig. 1B). Moreover, we performed immunohistochemical analysis of ZFX expression using HCC tissue microarrays (TMAs) containing 242 paired HCC specimens. An increased amount of ZFX was noticed in primary HCC samples as compared to their adjacent nontumorous tissues (Fig. 1C). We next investigated the clinical significance of ZFX expression in the progression of HCC. Based on the expression level of ZFX in TMAs, all 242 patients with HCC (Table S1) were divided into two groups: low‐ZFX expression group (*n* = 103) and high‐ZFX level group (*n* = 139). As shown in Table 1, the upregulation of ZFX in HCC samples was significantly associated with multiple malignant clinicopathological features of HCC, such as tumor size, tumor number, and tumor differentiation grade. More importantly, Kaplan–Meier analysis revealed that patients with higher ZFX expression exhibited much worse overall survival (OS) (*P* < 0.001) and recurrence‐free survival (RFS; *P* < 0.001) (Fig. 1D,E and Table 1). Taken together, ZFX is frequently enriched in human HCCs and may serve as a predictor for recurrence and poor survival of patients with HCC.

**Table 1:** Clinicopathological characteristics of HCC subtypes defined by ZFX expression

HCC subtypes	ZFX expression		*P* value^a^
	Low (*n* = 103)	High (*n* = 139)	
Sex			
Male	90	121	0.940
Female	13	18	
Age (year)			
> 60	23	26	0.488
≤ 60	80	113	
HBeAg‐positive			
Yes	103	138	1.000
No	0	1	
AFP (ng·mL^−1^)			
≥ 400	56	91	0.080
< 400	47	48	
Tumor size (cm)			
≥ 3	37	22	< 0.001
< 3	66	117	
Tumor number (count)			
≥ 2	12	33	0.017
1	91	106	
Tumor differentiation grade			
I–II	15	8	0.021
III–IV	88	131	
Tumor satellites			
Yes	78	102	0.679
No	25	37	
Microvascular invasion			
Yes	64	87	0.943
No	39	52	
Recurrence			
Yes	29	105	< 0.001
No	74	34	
Expired			
Yes	9	93	< 0.001
No	94	46	
Risk‐free survival time (months)^b^	55.3 ± 1.6	22.7 ± 2.1	< 0.001
Time of follow‐up (months)^b^	61.9 ± 0.9	32.7 ± 2.1	< 0.001

a Statistical significance was calculated by chi‐square test or Fisher's exact test for categorical/binary measures and ANOVA for continuous measures.

b Data are presented as mean ± SD.

### 3.2 Combination of ZFX and EpCAM expression is a significant prognostic factor in HCCs

**Figure 2:**
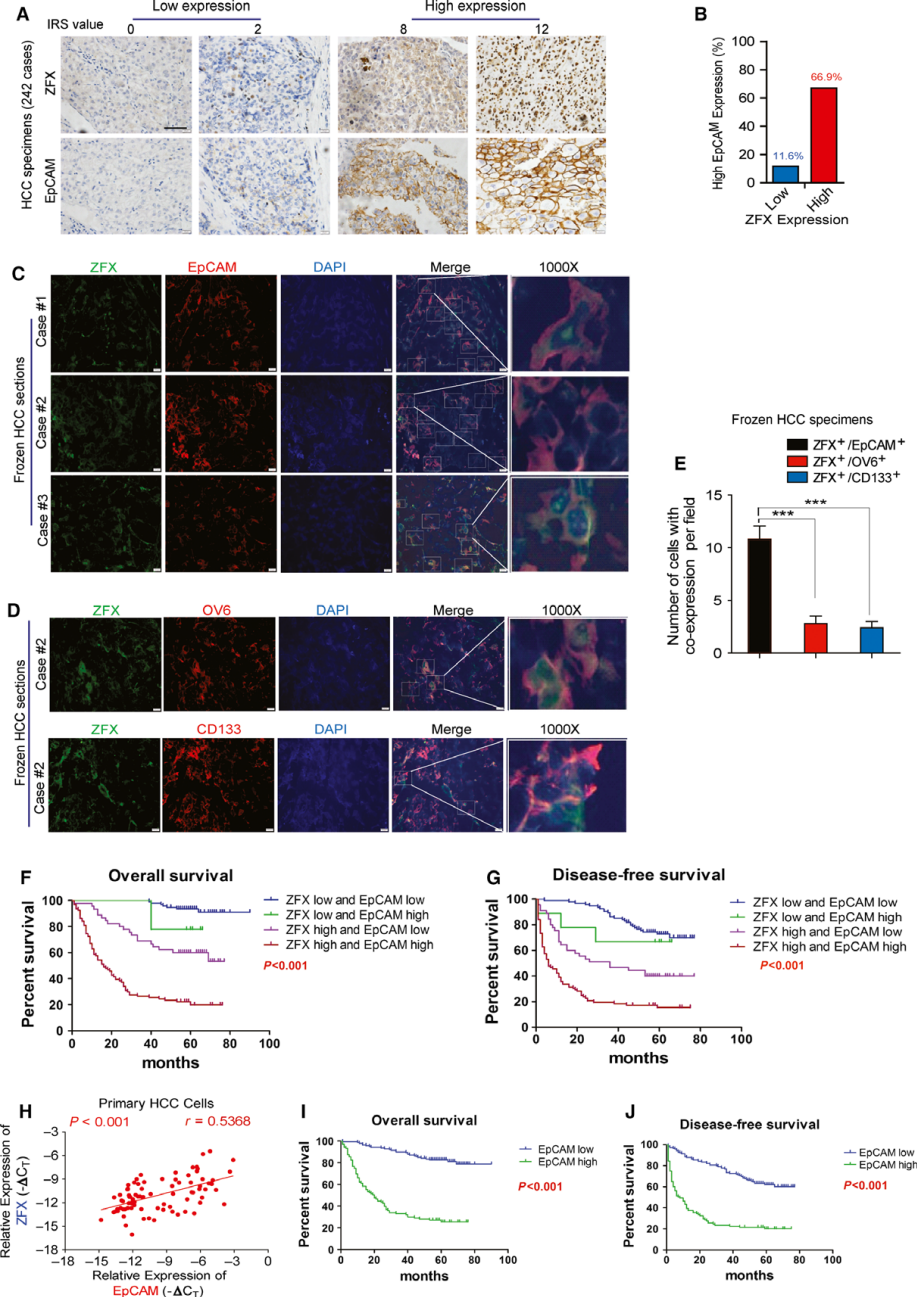
Combination of ZFX and EpCAM expression is a significant prognostic factor in HCCs. (A) HCC TMA sections were applied for IHC study on expression of ZFX and EpCAM (*n* = 242). Representative IHC staining of ZFX and EpCAM in HCC specimens is shown (scale bar = 50 μm). (B) The proportion of patients with relative high EpCAM staining was much higher in the ZFX‐high group (66.9%) when compared to that in the ZFX‐low group (11.6%). (C) Frozen sections of tumor tissues from different patients with HCC (Case #1, #2, #3) were stained with ZFX (green) and EpCAM (red). Nuclei were stained with DAPI dye, and amplified frames refer to ZFX^+^/EpCAM^+^ cells (scale bar = 50 μm). (D) Colocalization of ZFX and OV6 or CD133 in fresh frozen HCC sections. Representative pictures are shown and amplified frames refer to co‐expression cells (scale bar = 50 μm). (E) Under fluorescence microscope, five visual fields were randomly selected and number of indicated co‐expressed cells was counted per field. All data are presented as mean ± SD. ***P < 0.001. (F,G) The disease‐free survival (DFS) or overall survival (OS) rates of 242 patients with HCC were compared among different groups. (H) The mRNA expression levels of ZFX and EpCAM in mechanically dissociated primary HCC cells (69 cases) were evaluated by qRT‐PCR. The correlation analysis of ZFX and EpCAM expression in different HCCs was performed (Spearman *r* = 0.5368; *P* value < 0.001). (I,J) Kaplan–Meier analysis of the association between EpCAM expression levels and DFS or overall survival (OS) in patients with HCCs (*n* = 242).

Given the significance of CSCs in HCC recurrence and aggressive progression, we next explored the potential role of ZFX in regulating liver CSCs. A previous study has indicated EpCAM‐positive cells as putative liver CSC subpopulation in HCCs (Yamashita *et al*., 2009). To test whether ZFX expression correlates with the expansion of liver CSCs, we determined both the expression levels of ZFX and EpCAM in 242 patients with primary HCC using TMAs. As revealed by immunohistochemical (IHC) analysis, elevated levels of ZFX were mainly detected in HCC tissues with high EpCAM staining (Fig. 2A,B), indicating a positive relationship between ZFX and EpCAM expression. More importantly, EpCAM expression was commonly observed in ZFX‐expressing HCC cells from fresh frozen tumor sections by immunofluorescence staining, suggesting the co‐expression of ZFX and EpCAM within same cells of HCC specimens (Fig. 2C). To clarify whether ZFX is specifically highly expressed in EpCAM^+^ CSCs on patient specimens, we also detected the co‐expression of ZFX and other accepted CSC markers, such as CD133 and OV6, in HCC tissues. As illustrated in Fig. 2D, colocalization of ZFX and CD133 or OV6 were observed in same cells of a patient with HCC. Of note, in contrast to the frequent colocalized ZFX and EpCAM in HCC cells, only a small percentage of ZFX‐expressing HCC cells were found to co‐express CD133 or OV6 in same HCC tissues (Fig. 2E). Therefore, it was reasonable to suspect that ZFX is mainly expressed in EpCAM^+^ liver CSCs and related to their expansion in clinical samples.

Next, we investigated whether co‐expression of ZFX and EpCAM affected the malignant biological behaviors of HCCs. Based on the expression level of ZFX and EpCAM in HCC samples, all 242 patients were divided into four groups: I (*n* = 94), both high ZFX and EpCAM content; II (*n* = 45), high ZFX but low EpCAM content; III (*n* = 9), low ZFX but high EpCAM content; and IV (*n* = 94), both low ZFX and EpCAM content. Compared with other groups, the patients in ZFX^high^/EpCAM^high^ groups exhibited much more aggressive clinicopathological features such as AFP, tumor size, tumor number, and tumor differentiation grade (Table 2). More importantly, patients whose tumors both showed elevated expression of these two parameters often suffered worse OS (*P* < 0.001) (Fig. 2F) and exhibited shorter time to recurrence (*P* < 0.001) (Fig. 2G). In consistence with these clinical data, we also detected a significant relationship between ZFX and EpCAM levels in a cohort of fresh HCC biopsies (Fig. 2H). Particularly, we determined whether EpCAM could be an independent prognostic factor in HCC. According to the expression level of EpCAM in HCC samples, all 242 patients with HCC were divided into two groups: low‐EpCAM expression group (*n* = 139) and high‐EpCAM level group (*n *= 103). As shown in Table S2, EpCAM in HCC samples was significantly associated with tumor size and tumor number. In addition, Kaplan–Meier analysis revealed that patients with enhanced EpCAM expression exhibited much worse overall survival (OS; *P* < 0.001) and RFS (*P* < 0.001) (Fig. 2I,J and Table S2). In conclusion, EpCAM can serve as an independent prognostic factor in HCC. Furthermore, concomitant expression of ZFX and EpCAM is an effective prognostic predictor for HCC.

**Table 2:** Clinicopathological characteristics of HCC subtypes defined by ZFX and EpCAM expression

HCC subtypes	ZFX/EpCAM expression				*P* value^a^
	Both high (*n* = 94)	Either high		Both low (*n* = 94)	
		High ZFX, low EpCAM (*n* = 45)	High EpCAM, low ZFX (*n* = 9)		
Sex					
Male	82	39	7	83	0.762
Female	12	6	2	11	
Age (year)					
> 60	16	10	2	21	0.759
≤ 60	78	35	7	73	
HBeAg‐positive					
Yes	1	0	0	0	1.000
No	93	45	9	94	
AFP (ng·mL^−1^)					
≥ 400	59	32	8	48	0.033
< 400	35	13	1	46	
Tumor size (cm)					
≥ 3	80	37	6	60	0.004
< 3	14	8	3	34	
Tumor number (count)					
≥ 2	27	6	0	12	0.013
1	67	39	9	82	
Tumor differentiation grade					
I–II	7	1	0	15	0.044
III–IV	87	44	9	79	
Tumor satellites					
Yes	67	35	8	70	0.700
No	27	10	1	24	
Microvascular invasion					
Yes	59	28	6	58	1.000
No	35	17	3	36	
Recurrence					
Yes	78	27	3	26	< 0.001
No	16	18	6	68	
Expired					
Yes	74	19	2	7	< 0.001
No	20	26	7	87	
Risk‐free survival time (months)^b^	17 ± 2.2	34.7 ± 3.8	45.8 ± 8.3	56.2 ± 1.5	< 0.001
Time of follow‐up (months)^b^	25.2 ± 2.3	48.6 ± 3.3	56.8 ± 3.3	62.4 ± 1	< 0.001

a Statistical significance was calculated by chi‐square test or Fisher's exact test for categorical/binary measures and ANOVA for continuous measures.

b Data are presented as mean ± SD.

### 3.3 ZFX is predominantly enriched in liver CSCs

**Figure 3:**
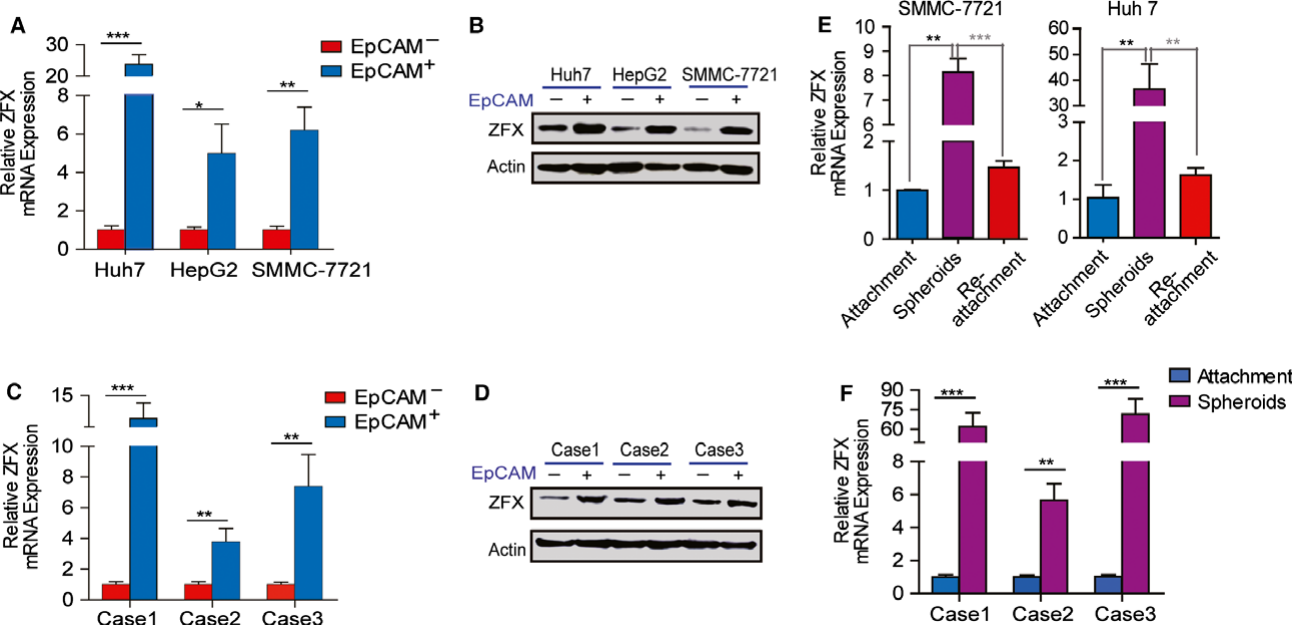
ZFX is preferentially enriched in liver CSCs. (A,B) EpCAM^+^ HCC cells were magnetically sorted from HCC cell lines (Huh7, HepG2, SMMC‐7721), and the mRNA and protein expression level of ZFX in EpCAM^+^ subpopulations and their counterparts was evaluated by qRT‐PCR and western blot. (C,D) EpCAM^+^ HCC cells were magnetically sorted from fresh HCC specimens (cases 1–3), and the mRNA and protein expression level of ZFX in EpCAM^+^ subpopulations and their counterparts was evaluated by qRT‐PCR and western blot. (E) ZFX expression of SMMC‐7721 and Huh7 cells maintained in different cultural conditions was analyzed by qRT‐PCR. (F) The isolated primary HCC cells from another three representative cases were used for tumor spheroids formation under nonadherent conditions. Quantitative RT‐PCR assay was performed to determine ZFX expression in both primary tumor spheroids and adherent HCC cells. All data are shown as mean ± SD. *P < 0.05, **P < 0.01, and ***P < 0.001.

As ZFX is required for maintenance of pluripotency and involved in the differentiation and development of ESCs, we also wondered whether ZFX is obviously elevated in liver CSCs. To address this issue, we determined the expression level of ZFX in EpCAM^+^ subpopulation sorted from cultured HCC cell lines (SMMC‐7721, HepG2, and Huh7) or primary liver cancer cells. As shown in Fig. 3A,B, ZFX expression was significantly upregulated in the subset of EpCAM^+^ HCC cells compared with matched EpCAM^−^ cells. Similarly results were obtained in sorted primary EpCAM^+^ HCC cells (Fig. 3C,D). Tumor spheroid formation assay is a commonly used approach to evaluate CSCs *in vitro*. Here, we found that the content of ZFX was further enhanced in nonattached hepatoma spheroids compared with the monolayer cultured cells (Fig. 3E). Of note, ZFX expression was rapidly restored to routine levels after seeding back into adherent cultural conditions (Fig. 3E). Consistently, increased amounts of ZFX were also detected in HCC spheroids relative to the attached primary HCC cells from patients (Fig. 3F), further suggesting a putative role of ZFX in the maintenance of liver CSCs.

### 3.4 ZFX is required to maintain stem cell‐like features of EpCAM^+^ liver CSCs

**Figure 4:**
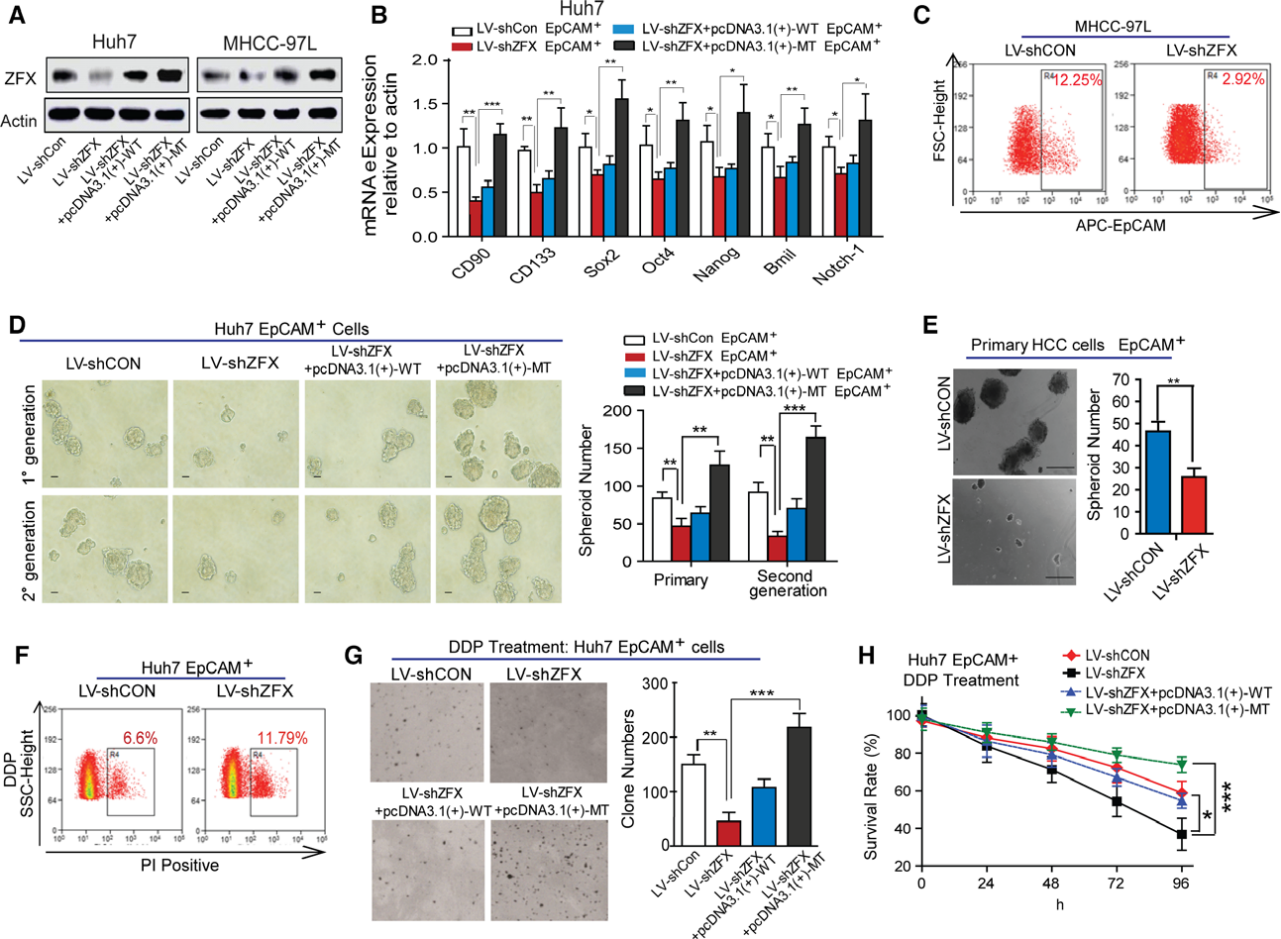
ZFX is required to maintain stem cell‐like features of EpCAM^+^ liver CSCs. (A) Expression of endogenous ZFX in MHCC‐97L and Huh7 cells with ZFX depletion (LV‐shZFX) or re‐expression (WT‐ or MT‐ZFX) were detected by western blot. (B) The mRNA expression levels of multiple stemness‐related genes in ZFX‐silenced or re‐expressed Huh7 EpCAM^+^ cells were evaluated by qRT‐PCR. Quantified mRNA levels were normalized to β‐actin and presented relative to the controls. (C) The percentage of EpCAM^+^ subpopulation in MHCC‐97L cells stably expressing shCON or shZFX was measured via flow cytometric assay. (D) Representative images of primary and secondary passaged HCC spheroids derived from EpCAM^+^ Huh7 cells with ZFX knockdown or re‐expression are shown (scale bar = 100 μm) (left panel), and spheroid number was counted. (E) Primary HCC cells sorted from resected HCC specimens were infected with LV‐shCON or LV‐shZFX, and EpCAM^+^ HCC cells were magnetically sorted for tumor spheroids formation assay. Representative images of HCC spheroids are shown (scale bar = 200 μm) (left panel). (F) Huh7 LV‐shCON EpCAM^+^ or LV‐shZFX EpCAM^+^ cells were treated with 2 μg·mL^−1^ cisplatin (DDP) for 4 days, and PI staining was performed to detect the proportion of dead cells via flow cytometry. (G) The colony formation capacity of EpCAM^+^ Huh7 cells with ZFX knockdown or re‐expression in the presence of 1 μg·mL^−1^ DDP for 2 weeks. Representative images of colonies are shown (left panel). (H) The indicated EpCAM^+^ HCC cells were treated with DDP (2 μg·mL^−1^), and cell viability was measured by CCK‐8 assay. Representative results from three independent experiments are shown, and all data represent mean ± SD, *P < 0.05, **P < 0.01, and ***P < 0.001.

To further explore the potential role of ZFX in regulating the stem‐like properties of EpCAM^+^ liver CSCs, we used a lentivirus‐based knockdown approach to establish shZFX‐stable transfectants of hepatoma cells (Huh7 and MHCC‐97L). The knockdown efficiency was verified by western blot and qRT‐PCR (Figs 4A and S1A). As shown in Figs 4B and S1B, EpCAM^+^ cells magnetic‐sorted from LV‐shZFX‐infected MHCC‐97L or Huh7 cells displayed decreased mRNA levels of several CSC markers (*CD90* and *CD133*) and a set of stemness‐related genes (including *Oct4*,* Nanog*,* Sox2*,* Bmil*, and *Notch‐1*). Consistently, flow cytometric assay also demonstrated that silencing of ZFX significantly reduced the percentage of EpCAM^+^ cells in total HCC cell pools (Figs 4C and S1C). As strengthened self‐renewal ability is one of the most important properties of CSCs, we conducted spheroid formation and *in vitro* limiting dilution assays to test whether ZFX could regulate the self‐renewal capacity of liver CSCs. Depletion of ZFX led to a decrease in both size and quantity of primary and serially passaged spheroids in EpCAM^+^ HCC cells (Figs 4D and S1D). Similarly, a decreased amount of tumor spheroids was observed in primary HCC cells infected with LV‐shZFX transfectants (Fig. 4E). The limiting dilution data further supported that knockdown of ZFX diminished the proportion of CSCs and attenuated their self‐renewal capacity *in vitro* (Table S3). As drug resistance of cancer stem cells is associated with chemotherapeutic failure and tumor relapse, we next determined whether ZFX could manipulate chemoresistance of liver CSC subsets. Using flow cytometric assay, we found that EpCAM^+^ CSCs sorted from LV‐shZFX‐infected Huh7 cells were more sensitive to cisplatin (DDP)‐induced cell death as compared to the control (Figs 4F and S1E). More importantly, depletion of ZFX in EpCAM^+^ liver CSCs obviously attenuated the chemoresistant colonies formation and impaired the cell survival rate during DDP treatment (Figs 4G,H and S1F–G). Additionally, to verify the potential role of ZFX in manipulating metastatic characteristics of liver CSCs, we also performed migration and Matrigel invasion assays. As expected, downregulation of ZFX apparently inhibited the invasive and migratory capacity of EpCAM^+^ liver CSCs when compared to that of control cells (Fig. S2). To test whether upregulation of ZFX could rescue these inhibitory effects, we re‐expressed either wide‐type (WT) or a codon‐optimized version mutant type (MT) of ZFX that was not targeted by shRNAs in shZFX‐infected HCC cell lines. As shown in Figs 4A and S1A, cells transfected with MT‐ZFX exhibited better overexpression effect. Subsequently, exogenous expression of ZFX enhanced the self‐renewal, chemoresistance, and migratory and invasive capacity of EpCAM^+^ transfected CSCs (Figs 4 and S1–S2). In conclusion, our findings suggest that ZFX is essential to expand EpCAM^+^ liver CSCs and maintain their stem‐like properties *in vitro*.

### 3.5 Depletion of ZFX inhibits tumorigenicity and pulmonary metastasis of EpCAM^+^ liver CSCs

**Figure 5:**
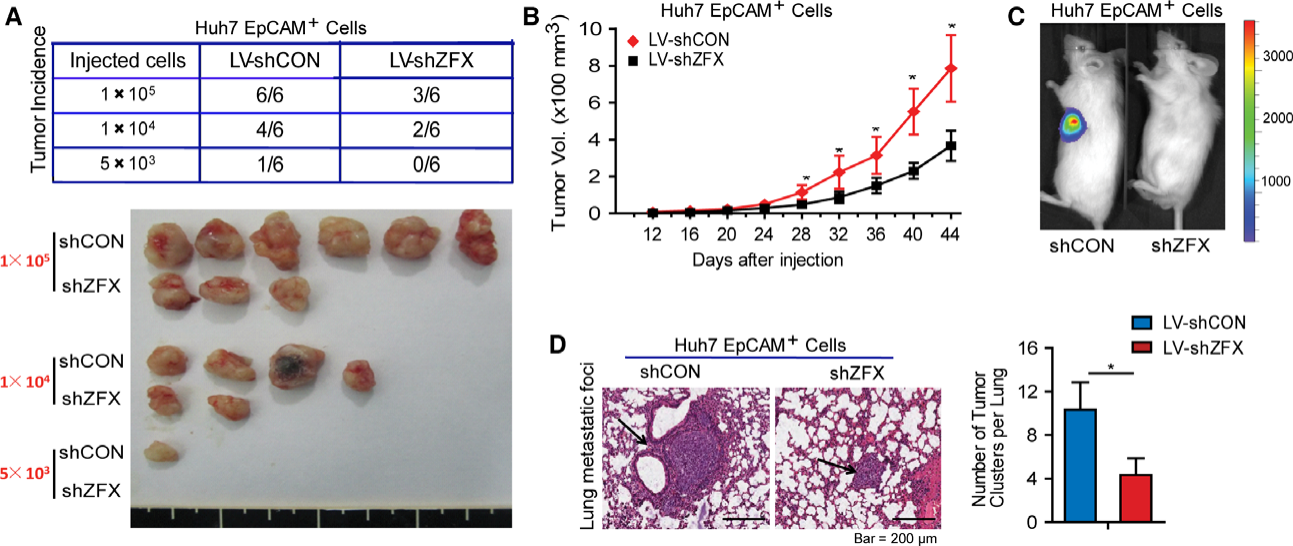
Silencing of ZFX inhibits tumorigenicity and pulmonary metastasis of EpCAM^+^ liver CSCs. (A) Mice were randomly divided into two groups and subcutaneously injected with indicated number of LV‐shCON‐ or LV‐shZFX‐infected Huh7 EpCAM^+^ cells, respectively. All mice were killed at day 44 and tumor incidence was evaluated (upper panel). Representative subcutaneous tumors are shown (lower panel). (B) Tumor volumes of xenografts from mice injected with 1 × 10^5^ LV‐shCON‐ or LV‐shZFX‐infected Huh7 EpCAM^+^ cells were monitored and calculated every four days. All data display mean ± SD, **P < 0.01. (C) 5 × 10^3^ LV‐shCON‐ or LV‐shZFX‐transfected Huh7 EpCAM^+^ cells were injected subcutaneously into NOD/SCID mice recipients and tumor formation was evaluated by bioluminescent imaging. (D) Representative sections of lung tissues from nude mice injected with 1 × 10^5^ LV‐shCON‐ or LV‐shZFX‐infected Huh7 EpCAM^+^ cells via tail vein are shown (left panel) (H&E staining, scale bar = 200 μm). Black arrows indicated lung metastatic foci. The number of lung metastatic tumors in each group (*n* = 6) was counted. All data represent mean ± SD, *P < 0.05.

Previous results have shown that EpCAM^+^ HCC cells were more prone to initiate tumors *in vivo* (Yamashita *et al*., 2009). To assess whether ZFX has a function in EpCAM^+^ CSC subset‐induced tumorigenicity and metastatic dissemination *in vivo*, we performed xenograft assays using a series of gradient EpCAM^+^ subpopulation sorted from Huh7‐LV‐shCON or shZFX cells. As shown in Fig. 5A, 1 × 10^5^ EpCAM^+^/shCON Huh7 cells efficiently generated large tumors in 100% of mice, but the equal numbers of EpCAM^+^/shZFX Huh7 cells produced only three tumors among six injected nude mice at 7 weeks after injection. Likewise, as little as 5 × 10^3^ EpCAM^+^/shCON cells could initiate tumors in one of six injected mice, whereas 5 × 10^3^ EpCAM^+^ cells expressing shZFX failed to do so at 7 weeks after transplantation, indicating that knockdown of ZFX weakened the EpCAM^+^ fraction‐driven tumorigenicity *in vivo*. In line with the tumor incidence between these two subpopulations, mice injected with EpCAM^+^/shZFX cells displayed progressive lower tumor burden than the mice transplanted with control cells (Fig. 5B). In addition, NOD/SCID mice bearing EpCAM^+^/shZFX cell‐derived xenografts demonstrated a significant decrease in bioluminescence (Fig. 5C), further confirming the attenuated tumorigenicity of EpCAM^+^ CSCs after ZFX depletion. Next, we established pulmonary metastatic models using sorted EpCAM^+^ Huh7 cells stably expressing shRNA targeting negative sequence or ZFX. As anticipated, fewer and smaller pulmonary metastatic foci were observed in mice transplanted with EpCAM^+^/shZFX Huh7 cells (Fig. 5D). Taken together, our data indicate that ZFX is capable of promoting the tumorigenicity and distant metastasis of EpCAM^+^ liver CSCs during HCC progression.

### 3.6 ZFX activates β‐catenin signaling via promoting β‐catenin nuclear translocation and accumulation

**Figure 6:**
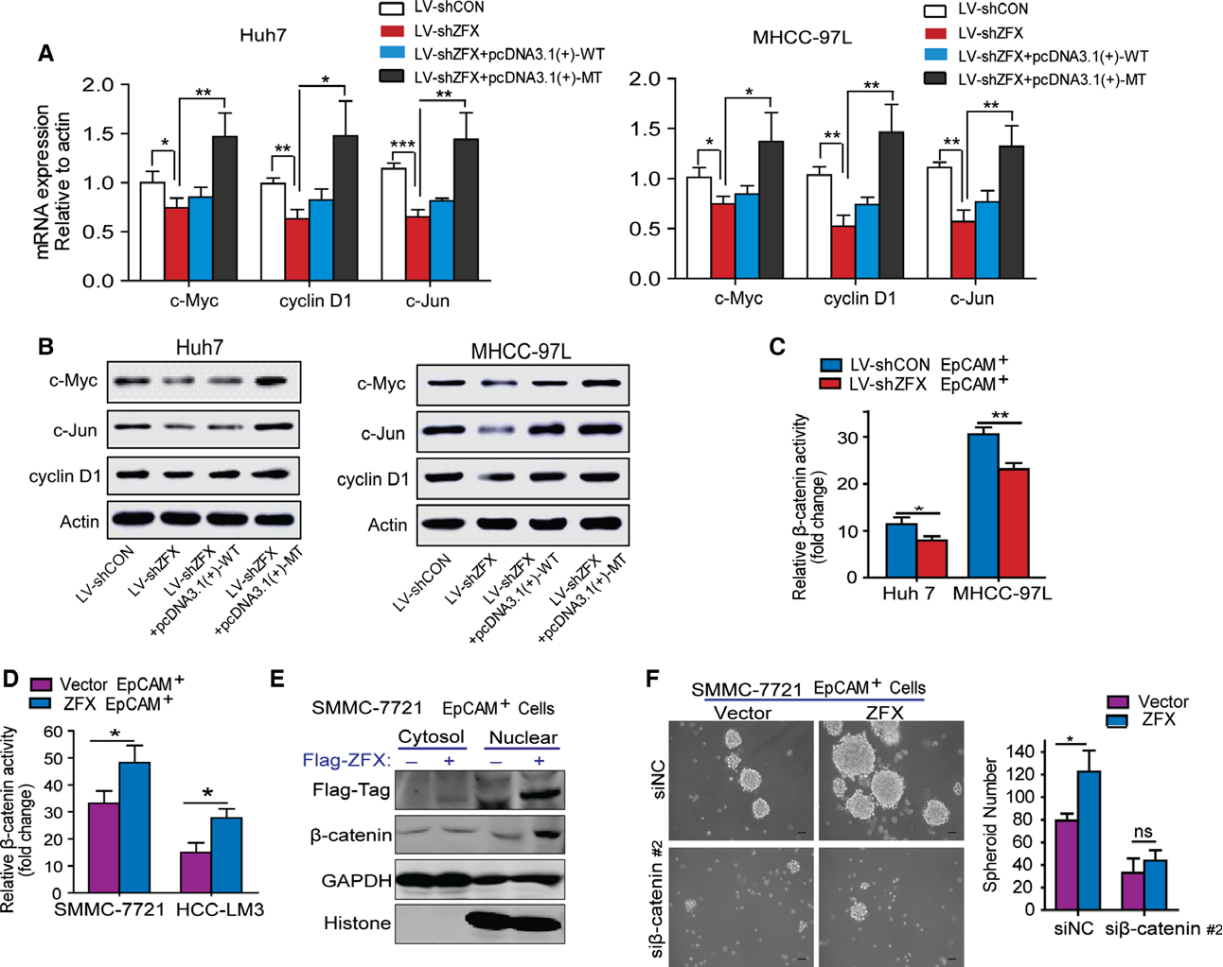
ZFX activates β‐catenin signaling via promoting its nuclear translocation and accumulation. (A) The mRNA levels of β‐catenin targeted genes in ZFX‐knockdown or ZFX‐overexpressed Huh7 and MHCC‐97L cells were measured by qRT‐PCR. (B) The protein levels of downstream targets of β‐catenin in ZFX‐knockdown or ZFX‐overexpressed Huh7 and MHCC‐97L cells were measured by western blot assay. (C) LV‐shCON‐ or LV‐shZFX‐infected Huh7 and MHCC‐97L EpCAM^+^ cells were transfected with β‐catenin luciferase reporter followed by luciferase assay. (D) SMMC‐7721 and HCC‐LM3 EpCAM^+^ cells stably expressing ZFX or empty vector were transfected with β‐catenin luciferase reporter followed by luciferase assay. (E) The cytoplasmic and nuclear protein of SMMC‐7721 EpCAM^+^ cells transfected with empty vector or Flag‐tagged‐ZFX plasmids were extracted, respectively, and subjected to western blotting assay. The antibodies were used as indicated. GAPDH and histone were used as internal loading control of cytoplasmic and nuclear protein, respectively. (F) SMMC‐7721 EpCAM^+^ cells expressing empty vector or ZFX were transfected with small interference RNAs (siRNA; siNC, and siβ‐catenin; sequence #2) targeting negative domain or endogenous β‐catenin, and capacity of tumor spheroid formation was evaluated. Representative images of HCC spheroids in different group are shown. In (A), (C), (D), and (F), experiments were performed in triplicate and all data represent mean ± SD, *P < 0.05, **P < 0.01, and ***P < 0.001.

We next investigated the underlying mechanisms by which ZFX maintained the stem‐like characteristics of EpCAM^+^ liver CSCs. Considering that aberrant activation of β‐catenin signaling pathway is pivotal for self‐renewal of liver CSCs and contributes to hepatocarcinogenesis (Cai and Zhu, 2012; Yamashita *et al*., 2007), we explored the possible relationship between ZFX and β‐catenin in our model. The western blot assay revealed that alternations of ZFX in HCC cells didn't impact β‐catenin expression level (Fig. S3A,B). Normally, the ubiquitylation and degradation of cytoplasmic β‐catenin is regulated by the intracellular ‘destructive complex’ which composed of adenomatous polyposis coli (APC), Axin‐1, and glycogen synthase kinase 3β (GSK‐3β) (Clevers and Nusse, 2012). However, both interference and overexpression of ZFX had no obvious effect on the expression levels of these key components which constituted the ‘β‐catenin degradation complex’ (Fig. S3A,B). Interestingly, we noticed that knockdown of ZFX suppressed the expression of several important β‐catenin target genes such as c‐Myc, c‐Jun, and cyclin D1 in hepatoma cells, whereas enforced expression of ZFX upregulated these downstream effectors both in mRNA and protein levels (Fig. 6A,B). These data suggest that ZFX participate to transcriptional activation of β‐catenin in nuclear. To test this hypothesis, we determined the effect of ZFX on β‐catenin transcriptional activity via luciferase reporter assays. As expected, silencing of ZFX attenuated the transcriptional activation of β‐catenin, as revealed by the decreased luciferase activity in shZFX‐stable EpCAM^+^ HCC cells compared with the control (Fig. 6C). Conversely, EpCAM^+^ HCC cells stably expressing ZFX exhibited elevated luciferase activity of β‐catenin (Fig. 6D). More importantly, forced ZFX expression enhanced the nuclear translocation of β‐catenin in the fraction of EpCAM^+^ cells (Fig. 6E), further indicating that ZFX augmented the transcription of β‐catenin signaling pathway and thus led to the expansion of putative CSCs. Moreover, blockage of β‐catenin by RNA‐interference strategy significantly abolished the capacity of tumor spheroid formation in those ZFX‐expressed EpCAM^+^ HCC cells (Fig. 6F). Collectively, our data indicate a critical role of ZFX in activating and maintaining liver CSCs by promoting the transport of β‐catenin into nucleus, where it induces transcription of downstream genes.

## 4 Discussion

In spite of recent therapeutic advances in the treatment of malignancies, current therapies for HCC remain largely ineffective because of highly acquired chemoresistance and rapidly tumor relapse (Aravalli *et al*., 2008). The growing understanding of malignant tumors has made it clear that CSCs play a critical role in the initiation and propagation of malignant tumors, including HCCs. Therefore, specific targeting of liver CSCs may repress the malignant biological behaviors and improve the curative effect. To this end, the underlying molecular mechanisms by which ZFX regulates the putative liver CSCs need further investigation. Here, we reported that ZFX is required for the activation and expansion of EpCAM^+^ liver CSCs. We first observed a positive correlation between ZFX and EpCAM expression in clinical HCC specimens. Similarly, ZFX was preferentially expressed in the fraction of EpCAM^+^ CSCs purified from cultured HCC cell lines. Moreover, disturbing ZFX by lentivirus‐based approaches significantly impaired the CSCs’ self‐renewal capacity and inhibited CSC‐driven HCC formation. Our further investigation also identified that Wnt/β‐catenin signaling was involved in the maintenance of liver CSCs. Mechanistically, forced ZFX expression promoted β‐catenin nuclear translocation and enhanced its transcriptional activity, thus contributing to the activation and expansion of liver CSCs. These findings also suggest that ZFX may exert as a therapeutic target aiming at CSCs eradication in HCC management.

Recent study on ZFX underlined a crucial role for this transcript factor in the maintenance of ESCs (Galan‐Caridad *et al*., 2007). In this model, increased level of ZFX contributed to the upregulation of ESC‐specific self‐renewal regulators Tbx3 and Tcl1 by directly binding to their promoter regions in undifferentiated ESCs, thus promoting ESC self‐renewal but not affect their differentiation. In addition, Chen *et al*. (2008) showed that ZFX could bind to loci occupied by a panel of core regulators including Oct4, Sox2, and Nanog, thus forming the multiple transcription factor binding loci. Therefore, this indirect involvement of ZFX might augment the functions of these regulatory elements in maintaining the self‐renewal of ESCs. Meanwhile, ZFX is required for the growth and differentiation of multiple cell types, including human ESCs, myeloid progenitors, and embryonic fibroblasts (Harel *et al*., 2012). Although CSCs are distinct from ESCs, it is undeniable that CSCs and ESCs share similar stem‐like properties. Therefore, it is plausible for us to speculate that ZFX may regulate the stemness‐related features of CSCs. Indeed, several studies have demonstrated the contribution of ZFX in the maintenance of stem cell‐like characteristics in multiple cancer types. Lai *et al*. (2014) found that ZFX induced the expression of stem‐like promoting genes, Nanog and Sox2, by directly binding on the promoter regions of these genes, thus conferring the self‐renewal and drug resistance properties in HCCs. Another group reported that ZFX could bind to a certain sequence on human c‐Myc promoter to upregulate its expression, thus maintaining the stem‐like phenotypes and tumorigenic potential of glioma stem cells (Fang *et al*., 2014). In the present study, we demonstrated that ZFX was preferentially expressed by EpCAM^+^ CSCs in human HCCs. To date, it is widely accepted that EpCAM is one of the most predominant biomarkers of liver CSCs. In consistent with the increased levels in EpCAM^+^ CSCs, ZFX expression levels were also correlated with the expression of those putative liver CSCs biomarkers. Considering that spheroid culture of cancer cells is a widely used approach to enrich and assess CSCs, we thereby determined the influence of ZFX on tumor spheroid formation. As expected, the increased amount of ZFX in isolated primary HCC spheroids further supported our hypothesis that ZFX accelerates HCC development via the regulatory effects on liver CSCs. In contrast, targeting of ZFX in magnetically sorted EpCAM^+^ CSCs inhibited their stemness phenotypes and attenuated their capacity of tumorigenesis *in vivo*. Of note, the malignant transformation of non‐CSCs has been proven to be involved in the formation of CSC subpopulation and contribute to brain tumor progression (Flavahan *et al*., 2013). However, little is known whether overexpression of ZFX is implicated in the malignant transformation of non‐CSCs to putative liver CSCs.

A previous study has reported that a series of signaling pathways are involved in modulating the biological functions and features of stem‐like cells (Scoville *et al*., 2008). Among them, aberrant activation of Wnt/β‐catenin signaling pathway is commonly detected in HCCs and plays a predominant role in mediating liver CSCs transformation and expansion during HCC formation (Fodde and Brabletz, 2007; Thompson and Monga, 2007; Yamashita *et al*., 2007). Concrete evidence has revealed that the expression of c‐Myc, the major downstream effector of β‐catenin pathway, was closely correlated with the stemness regulation, whereas inactivation of c‐Myc reversed the tumorigenic potential of CSCs (Shachaf *et al*., 2004). In the present study, we observed that silencing of ZFX suppressed the mRNA expression of c‐Myc, cyclin D1, and c‐Jun, which are the major target genes of β‐catenin signaling. Briefly, cytoplasmic β‐catenin can be rapidly phosphorylated by the so‐called destructive complex and consequently degradated by the ubiquitin/proteasome system (Clevers and Nusse, 2012). Thus, disturbing the formation of this complexus may lead to accumulation of stabilized β‐catenin in the cytoplasm, which is transported into the nucleus to induce transcription of downstream genes such as c‐Myc and cyclin D1. Of note, a current study has demonstrated that ZFX upregulated c‐Myc expression in glioma stem cells by binding to a specific sequence on the promoter region of c‐Myc (Fang *et al*., 2014). Although ZFX could directly transcriptional activated c‐Myc expression in the fraction of glioma stem‐like cells, our current data reveal a novel molecular mechanism by which ZFX controls c‐Myc expression in HCCs. We hereby noticed that forced ZFX expression facilitated the nuclear translocation of β‐catenin and thus enhanced its accumulation in HCC cells, which further promoted the transcriptional activation of downstream target genes. To our knowledge, this is the first report concerning the ZFX‐driven regulations on β‐catenin signaling cascades in liver CSCs. Even so, we still have no idea how ZFX regulates the stability of β‐catenin in cytoplasm. To date, no clear evidence has elucidated how ZFX influences any components of the destructive complex or β‐catenin itself. Likewise, our co‐immunoprecipitation assay failed to detect a direct interaction between β‐catenin and ZFX in both liver CSC or non‐CSC subsets. Moreover, the alternation of ZFX contents did not impact the expression levels of APC, Axin‐1, and GSK‐3β in HCCs. Therefore, the detail mechanism underlying the impacts of ZFX in β‐catenin stabilization still needs further exploration.

Based on our aforementioned results, we believed that ZFX contributes to the invasiveness and metastasis of HCCs, at least to some extent, through its modulation on the fraction of liver CSCs. Our notion was further supported by the clinical investigation which revealed that ZFX combined with EpCAM may provide a novel prognostic indicator for patients with HCC. In summary, our data suggest that interference of ZFX could be exploited for therapeutic benefit and represent an effective adjuvant anticancer treatment to eliminate CSCs during HCC progression.

## Data accessibility

Research data pertaining to this article is located at figshare.com: https://dx.doi.org/10.6084/m9.figshare.5002088.

## Author contributions

CW, SF, and MW contributed to study concept, design, and acquisition of data. WY, QC, HH, WD, HW, and YY contributed to acquisition of data. WZ and PL contributed to analysis and interpretation of data. WD contributed to statistical analysis. MW contributed to critical revision of the manuscript. WZ contributed to study concept and design, drafting of the manuscript, and critical revision of the manuscript.

## Supporting information


**Table S1.** Summary of clinicopathological variables.
**Table S2.** Clinicopathological characteristics of HCC subtypes defined by EpCAM expression.
**Table S3.** *In vitro* limiting dilution assay.
**Table S4.** Sequence information of wide‐type and mutant‐type ZFX.
**Table S5.** Sequence of siRNAs targeting β‐catenin in this study.
**Table S6.** Sequence of PCR primers used in this study.
**Fig. S1.** ZFX is required to maintain stem cell‐like features of EpCAM^+^ liver CSCs (related to Figure 4).
**Fig. S2.** The impact of ZFX on EpCAM^+^ HCC cell invasion and migration *in vitro* (related to Figure 4).
