# Supplementary material for: Zinc finger protein X‐linked promotes expansion of EpCAM+ cancer stem‐like cells in hepatocellular carcinoma
Source: Mol Oncol. 2017 Mar 29;11(5):455–69. doi: 10.1002/1878-0261.12036 (PMC5527465; doi:10.1002/1878-0261.12036)
Supplement: Supplementary file 1 — Table S1. Summary of clinicopathological variables. Table S2. Clinicopathological characteristics of HCC subtypes defined by EpCAM expression. Table S3. In vitro limiting dilution assay. Table S4. Sequence information of wide‐type and mutant‐type ZFX. Table S5. Sequence of siRNAs targeting β‐catenin in this study. Table S6. Sequence of PCR primers used in this study. Fig. S1. ZFX is required to maintain stem cell‐like features of EpCAM+ liver CSCs (related to Figure 4). Fig. S2. The impact of ZFX on EpCAM+ HCC cell invasion and migration in vitro (related to Figure 4). [file MOL2-11-455-s001.docx]

**Zinc finger protein X-linked promotes expansion of EpCAM^+^ cancer stem like cells in hepatocellular carcinoma**

Chao Wang^†1,2^ , Si-yuan Fu^†^^1^, Ming-da Wang^†3^, Wen-bo Yu^4^, Qin-shu Cui^4^, Hong-ru Wang^4^, Hai Huang^5^, Wei Dong^6^, Wei-wei Zhang^7^, Peng-peng Li^1^, Chuan Lin^1^, Ze-ya Pan^1^, Yuan Yang^1^, Meng-chao Wu^3^ and Wei-ping Zhou*^1^.

**Authors address**

1 The Third Department of Hepatic Surgery, Eastern Hepatobiliary Hospital, Second Military Medical University, 225 Changhai Road, Shanghai 200438, China.

2 Department of Urology, Changhai Hospital, Second Military Medical University, 168 Changhai Road, Shanghai 200438, China.

3 The Department of Hepatic Surgery, Eastern Hepatobiliary Hospital, Second Military Medical University, 225 Changhai Road, Shanghai 200438, China.

4 The Second Military Medical University, 800 Xiangyin Road, Shanghai 200433, China.

5 Department of Urinary Surgery, Changzheng Hospital, Second Military Medical University, No. 415 Feng Yang Road, Shanghai 200003, China.

6 Data Scientist, Liberty Mutual Group, 157 Berkeley Street, Boston, Massachusetts 02116, USA.

7 Department of Laboratory Diagnostic, Changhai Hospital, Second Military Medical University, 168 Changhai Road, Shanghai 200438, China.

† These authors contributed equally to this work.

* Corresponding authors. **Address correspondence to:** The Third Department of Hepatic Surgery, Eastern Hepatobiliary Hospital, Second Military Medical University, 225 Changhai Road, Shanghai 200438, China. Tel.: +86 21 81875521; fax: +86 02181875529. E-mail: ehphwp@126.com (W.-P. Zhou).

**Supplementary Data**

**Part 1. Materials and methods**

***Cell lines, gene silencing and plasmid transfection***

HCC cell lines Huh7, MHCC-97L, HepG2, SMMC-7721, and HCC-LM3 were obtained from Cell Bank of Type Culture Collection of the Chinese Academy of Sciences. Cell lines were routinely cultured in Dulbecco’s modified Eagle’s medium (Gibco) supplemented with 10% fetal bovine serum (FBS). Stable Huh7 and MHCC-97L cells silenced for ZFX were generated using lentiviral constructs expressing shZFX and negative control (Genechem Co., Shanghai), and incubated with 2 μg/mL puromycin (Sigma-Aldrich). For the rescue studies *in vitro*, a codon optimized version of ZFX (non-targeted by shZFX) was designed by Genechem Co. (Shanghai). Briefly, shZFX targeted sequence “GTCGGAAATTGATCCTTGTAA” in ZFX cDNA was mutated to “GAGCGAGATCGACCCCTGCAA”, and both the wide-type (WT) and mutant-type (MT) of human ZFX cDNA were constructed into pcDNA3.1(+) vectors. The sequence information of ZFX plasmids was listed in Supplementary Table 4. Then the shZFX-HCC cells were transfected with plasmids containing WT or MT-ZFX according to the manufacturer’s instructions. For ZFX overexpression, SMMC-7721 and HCC-LM3 cells were transfected with pCMV6-empty vector or pCMV6-ZFX (BO YI Biotechnology Co., Ltd, Shanghai) by using Lipofectamine^TM^ 2000 reagent (Invitrogen, Carlsbad, CA). After 48 hour post-transfection, cells were incubated in medium with 500 mg/ml Geneticin G418 (Sigma-Aldrich) and selected for 3 weeks. The small interference RNAs (siRNAs) for β-catenin and negative control were purchased from Ribobio Co. (Guangzhou, China). The sequences of siRNAs against β-catenin are shown in **Supplementary Table 5**. The transient transfection of siRNAs was performed with Lipofectamine^TM^ 2000 reagents according to manufacturer’s instructions. The original medium was replaced with fresh medium containing 10% FBS 12 hours post transfection.

***Real-time PCR and Western blotting***

Total RNA from different cell lines, isolated primary HCC cells, and human tissues was extracted using TRIzol reagent (Gibco, Carlsbad, CA). Quantitative RT-PCR was performed using an ABI 7300 Fast Real-Time PCR System (Applied Biosystems, CA, USA) and SYBR Green PCR kit (Applied TaKaRa, Shiga, Japan). The primer sequences were presented in **Supplementary Table 6**. Each measurement was performed in triplicate and the results were normalized by the expression of the β-actin gene. Fold change relative to mean value was determined by 2**^-△△^**^Ct^. The cell lyses of indicated HCC cells or human HCC tissues were analyzed by western blot using Odyssey fluorescence scanner and LI-COR imaging system (Li-COR Biosciences, Lincoln, NE). The primary antibodies used were listed as following: anti-ZFX, anti-c-Myc, anti-c-Jun (Abcam, Cambridge, MA), anti-Flag-tag, anti-β-catenin, anti-Histone, anti-GSK3β, anti-Axin1, anti-cyclin D1 (Cell Signaling Technology, Danvers, MA), anti-APC, anti-GAPDH, and anti-β-actin (Santa Cruz Biotechnology, Santa Cruz, CA).

***Immunohistochemistry***

Tissue microarray slides were incubated with the following primary antibodies at 4℃ overnight: anti-ZFX (1:80; Cell Signaling Technology, USA), and anti-EpCAM (1:100; Epitomics). Corresponding secondary antibodies were used, and diaminobenzidine (DAB) (Dako, Carpinteria, CA) staining was applied. Then the sections were counterstained with hematoxylin. The staining levels of ZFX and EpCAM in all clinical samples were examined by two independent observers, and according to the immunoreactive score (IRS) [[1](#_ENREF_1)]. Specially, the percentage of positive cells (% of PPs) and the staining intensity (SI value) were determined and multiplied (IRS value), and the score range is from a minimum score of 0 to a maximum score of 12. An IRS value more than one was considered as positive (weak expression); an IRS value more than three, moderate expression; and an IRS value more than eight, strong expression. Finally, patients with HCC were divided into: low expression (IRS value 0-3including negative and weak expression) and high expression (IRS value 4-12 including moderate and strong expression).

***Immunofluorescence staining***

Frozen sections of fresh human HCC tissues were stained with Rabbit anti-ZFX (Cell Signaling Technology, USA), anti-EpCAM, anti-CD133 (Abcam, Cambridge, MA) and Mouse anti-OV6 (R&D Systems, Minneapolis, MN) primary antibodies, followed by fluorescent staining with Alexa Fluor 555-conjugated IgG (Invitrogen) and Alexa Fluor 488-conjugated immunoglobulin G (IgG). Nuclear staining was conducted by 4,6-diamidino-2-phenylindole (DAPI). Representative images were captured with an Olympus IX70. Under the fluorescence microscope, five visual fields were randomly selected and number of those indicated co-expressed cells was counted per field.

***Spheroid formation assay***

For both HCC cell lines and freshly isolated primary HCC cells, single-cell suspensions of magnetic sorting EpCAM^+^ cells were incubated in 6-well ultra-low attachment culture microplates (Corning, NY, USA) for 14 days. The number of spheroids formed was counted under a microscopy 14 days after seeding and the representative pictures were taken.

***In vitro limiting dilution assay***

The in vitro extreme limiting assay was conducted by using ultra-low attachment culture dishes, as previously described [[2](#_ENREF_1)]. Briefly, indicated HCC cells were seeded into 96-well ultra-low attachment culture dishes at various cell numbers and incubated under spheroid conditions for 7 days. The number of colonies in each well was counted by visual inspection. According to the frequency of wells without colony, proportion of liver CSCs was evaluated using Poisson distribution statistics and the L-Calc Version 1.1 software program (Stem Cell Technologies, Inc.).

***Colony formation assay***

800 viable indicated HCC cells were seeded in 6-well plates for 10 days. Chemotherapy drug DDP (0.5 μg/ml) was added at the onset of incubation for 10 days. Then, the wells were washed with PBS buffer for twice and colonies were fixed with methanol and stained with methylene blue. The number of colonies was counted under a microscope and the representative views were photographed.

***Matrigel invasion and migration assay***

Invasion and migration assays were carried out in BioCoat Matrigel invasion chambers (BD Biosciences, USA) and transwell filter champers (Costar, Corning, NY) according to manufacturer's instructions. Briefly, 2 × 10^4^ cells cells were placed into the upper chamber and 10% FBS in DMEM was added to the bottom chamber as a chemoattractant. After 48-hour incubation at 37 °C, the number of cells that invaded through the Matrigel or membrane were photographed and counted after staining with 0.1% crystal violet for 15 min. Five random microscopic fields were selected for counting invaded cell numbers each group, and the experiments were repeated independently 3 times.

***Cell survival rate assay***

The cell survival rate assay was carried out using cholecystokinin-8 (CCK-8) (Dojindo, Japan) as previously described [[3](#_ENREF_2)]. Briefly, 6000 viable cells were seeded in triplicates in 96-well plates and DDP (0.5 μg/ml) was added at the beginning of culture period for 4 days. At indicated time points, each well was mixed with 10 μl CCK-8 and maintained for another 1 hour. Then, the OD values were measured using a microplate reader (Synergy HT, USA) at an absorbance of 450 nm. The survival rates were presented as a proportion of the control value which was detected at the first time point.

***Luciferase reporter assay***

A dual-luciferase reporter assay was performed using wild-type TCF-luciferase construct (pGL3-OT) and the mutant TCF-luciferase reporter construct (pGL3-OF). Indicated HCC cells were cultured in 24-well plates. After 24 hours, each well were transfected by lipofectamine 2000 (Invitrogen, Carlsbad, USA) with pGL3-OT or pGL3-OF. Meanwhile, all wells were co-transfected with pRL-TK plasmids which contained the Renilla luciferase genes as internal control for normalizing transfection efficiency. Then the luciferase activity was determined using the Promega luciferase assay reagent and Synergy 2 Multi-Detection Microplate Reader after 48 hours of incubation. The relative activity of β-catenin was presented as the OT/OF ratio [[4](#_ENREF_3)].

**References**

[1] [Tur MK](https://www.ncbi.nlm.nih.gov/pubmed/?term=Tur%20MK%5BAuthor%5D&cauthor=true&cauthor_uid=23462508), [Etschmann B](https://www.ncbi.nlm.nih.gov/pubmed/?term=Etschmann%20B%5BAuthor%5D&cauthor=true&cauthor_uid=23462508), [Benz A](https://www.ncbi.nlm.nih.gov/pubmed/?term=Benz%20A%5BAuthor%5D&cauthor=true&cauthor_uid=23462508), [Leich E](https://www.ncbi.nlm.nih.gov/pubmed/?term=Leich%20E%5BAuthor%5D&cauthor=true&cauthor_uid=23462508), [Waller C](https://www.ncbi.nlm.nih.gov/pubmed/?term=Waller%20C%5BAuthor%5D&cauthor=true&cauthor_uid=23462508), [Schuh K](https://www.ncbi.nlm.nih.gov/pubmed/?term=Schuh%20K%5BAuthor%5D&cauthor=true&cauthor_uid=23462508), et al. The 140-kD isoform of CD56 (NCAM1) directs the molecular pathogenesis of ischemic cardiomyopathy.[Am J Pathol.](https://www.ncbi.nlm.nih.gov/pubmed/?term=PMID%3A+23462508) 2013 Apr;182(4):1205-18.

[2] Wu K, Ding J, Chen C, Sun W, Ning BF, Wen W, et al. Hepatic transforming growth factor beta gives rise to tumor-initiating cells and promotes liver cancer development. Hepatology 2012;56:2255-2267.

[3] Zhang JW, Zhang SS, Song JR, Sun K, Zong C, Zhao QD, et al. Autophagy inhibition switches low-dose camptothecin-induced premature senescence to apoptosis in human colorectal cancer cells. Biochemical pharmacology 2014;90:265-275.

[4] Ning BF, Ding J, Yin C, Zhong W, Wu K, Zeng X, et al. Hepatocyte nuclear factor 4 alpha suppresses the development of hepatocellular carcinoma. Cancer research 2010;70:7640-7651.

**Part 2. Supplementary tables**

| **Supplementary Table 1. Summary of Clinicopathologic Variables** | |
| --- | --- |
| **Characteristic** | **No. of patients** |
| **Patient** | 242 |
| **Sex** |  |
| Male | 211 |
| Female | 31 |
| **Age (year)** |  |
| > 60 | 49 |
| ≤ 60 | 193 |
| **HBeAg** |  |
| Yes | 1 |
| No | 241 |
| **AFP (ng/ml) ^a^** |  |
| ≥ 400 | 147 |
| < 400 | 95 |
| **Tumor size (cm)** |  |
| ≥ 3cm | 183 |
| < 3cm | 59 |
| **Tumor number (count)** |  |
| ≥ 2 | 45 |
| 1 | 197 |
| **Tumor differentiation grade** | |
| I - II | 23 |
| III - IV | 219 |
| **Tumor satellites** |  |
| Yes | 180 |
| No | 62 |
| **Microvascular invasion** |  |
| Yes | 151 |
| No | 91 |
| **Recurrence** |  |
| Yes | 134 |
| No | 108 |
| **Expired** |  |
| Yes | 102 |
| No | 140 |
| **Risk-free Survival time (mo)** | 1 - 77 (median, 43.5) |
| **Time of folow-up (mo)** | 1 - 90 (median 55.5) |
| ^a^ AFP, serum -fetoprotein. | |

| **Supplementary Table 2. Clinicopathologic Characteristics of HCC Subtypes**  **Defined by EpCAM Expression** | | | |
| --- | --- | --- | --- |
|  | **EpCAM expression** | |  |
| **HCC subtypes** | **Low (n=139)** | **High (n=103)** | **P value^a^** |
| **Sex** |  |  |  |
| Male | 122 | 89 | 0.754 |
| Female | 17 | 14 |  |
| **Age (year)** |  |  |  |
| > 60 | 31 | 18 | 0.356 |
| ≤ 60 | 108 | 85 |  |
| **HBeAg positive** |  |  |  |
| Yes | 0 | 1 | 0.244 |
| No | 139 | 102 |  |
| **AFP (ng/ml)** |  |  |  |
| ≥ 400 | 80 | 67 | 0.238 |
| < 400 | 59 | 36 |  |
| **Tumor size (cm)** |  |  |  |
| ≥ 3cm | 97 | 86 | **0.014** |
| < 3cm | 42 | 17 |  |
| **Tumor number (count)** |  |  |  |
| ≥ 2 | 18 | 27 | **0.009** |
| 1 | 121 | 76 |  |
| **Tumor differentiation grade** |  |  |  |
| I - II | 16 | 7 | 0.216 |
| III - IV | 123 | 96 |  |
| **Tumor satellites** |  |  |  |
| Yes | 105 | 75 | 0.631 |
| No | 34 | 28 |  |
| **Microvascular invasion** |  |  |  |
| Yes | 86 | 65 | 0.844 |
| No | 53 | 38 |  |
| **Recurrence** |  |  |  |
| Yes | 53 | 81 | **< 0.001** |
| No | 86 | 22 |  |
| **Expired** |  |  |  |
| Yes | 26 | 76 | **< 0.001** |
| No | 113 | 27 |  |
| **Risk-free Survival time (mo)^b^** | 49.2 ± 1.8 | 19.5 ± 2.3 | **< 0.001** |
| **Time of folow-up (mo)^b^** | 57.9 ± 1.3 | 27.9 ± 2.3 | **< 0.001** |
| ^a^ Statistical significance was caluculated by chi-square test or fisher's exact test for categorical/binary measures and ANOVA for continuous measures.  ^b^ Data are presented as mean ± SD. | | | |

**Supplementary Table 3. *In vitro* Limiting Dilution Assay**

|  | **Number of Cells Seeded Each Well** | | | | | |  | **Stem Cell Frequency** | |  | | **Statistics** | | |  |
| --- | --- | --- | --- | --- | --- | --- | --- | --- | --- | --- | --- | --- | --- | --- | --- |
| **Huh7 EpCAM^+^** | **32** | **16** | **8** | **4** | **2** | **1** |  | **Estimate** | **Upper and Lower Limits** |  | | **Ratio of Prop.'s^#^** | **P Value^*^** | **ref. group** |  |
| Huh7 EpCAM^+^ shCon | 8/8 | 6/8 | 4/8 | 2/8 | 2/8 | 1/8 |  | 1:10.1 | (1:6.3 - 1:16.1) |  | | - | - | - |  |
| Huh7 EpCAM^+^ shZFX | 6/8 | 4/8 | 2/8 | 1/8 | 0/8 | 0/8 |  | 1:26.4 | (1:15.2 - 1:45.9) |  | | 2.61 | **0.009** | Huh7 EpCAM^+^ shCon |  |
| Huh7 EpCAM^+^ shZFX-pcdna3.1(+)-WT | 7/8 | 5/8 | 3/8 | 2/8 | 0/8 | 0/8 |  | 1:17.4 | (1:10.6 - 1:28.7) |  | | - | - | - |  |
| Huh7 EpCAM^+^ shZFX-pcdna3.1(+)-MT | 8/8 | 8/8 | 6/8 | 5/8 | 3/8 | 2/8 |  | 1:4.4 | (1:2.7 - 1:7) |  | | 0.17 | **< 0.001** | Huh7 EpCAM^+^ shZFX |  |
|  | | | | | | | | | | | | | | | |
|  | **Number of Cells Seeded Each Well** | | | | | |  | **Stem Cell Frequency** | |  | **Statistics** | | | |  |
| **MHCC-97L EpCAM^+^** | **32** | **16** | **8** | **4** | **2** | **1** |  | **Estimate** | **Upper and Lower Limits** |  | **Ratio of Prop.'s^#^** | | **P Value^*^** | **ref. group** |  |
| MHCC-97L EpCAM^+^ shCon | 8/8 | 7/8 | 4/8 | 3/8 | 2/8 | 1/8 |  | 1:8.4 | (1:5.3 - 1:13.3) |  | - | | - | - |  |
| MHCC-97L EpCAM^+^ shZFX | 6/8 | 3/8 | 2/8 | 1/8 | 0/8 | 0/8 |  | 1:29.5 | (1:16.7 - 1:52.3) |  | 3.54 | | **< 0.001** | MHCC-97L EpCAM^+^ shCon |  |
| MHCC-97L EpCAM^+^ shZFX-pcDNA3.1(+)-WT | 6/8 | 4/8 | 3/8 | 2/8 | 0/8 | 0/8 |  | 1:22.2 | (1:13.1 - 1:37.6) |  | - | | - | - |  |
| MHCC-97L EpCAM^+^ shZFX-pcDNA3.1(+)-MT | 8/8 | 8/8 | 7/8 | 5/8 | 4/8 | 2/8 |  | 1:3.5 | (1:2.2 - 1:5.7) |  | 0.12 | | **< 0.001** | MHCC-97L EpCAM^+^ shZFX |  |

**^#^** Ratio of Prop.'s compared the frequency estimates between HBx and Con groups.

^*^ Statistical significance was calculated by overall test for differences in stem cell frequencies between two groups (Chi-square test).

| **Supplementary Table 4. Sequence Information of Wide-type and Mutant-type ZFX** | |
| --- | --- |
| Wide-type ZFX | |
|  | *>gi\|296010876:308-2725 Homo sapiens zinc finger protein, X-linked (ZFX), transcript variant 1, mRNA*  ATGGATGAAGATGGGCTTGAATTACAACAAGAGCCAAACTCATTTTTTGATGCAACAGGAGCTGATGGTACACACATGGATGGTGATCAAATTGTTGTGGAAGTACAAGAAACTGTTTTTGTTTCAGATGTTGTGGATTCAGACATAACTGTGCATAACTTTGTTCCTGATGACCCAGATTCAGTTGTAATCCAAGATGTTATTGAGGACGTTGTTATAGAAGATGTTCAGTGCCCAGATATCATGGAAGAAGCAGATGTGTCTGAAACGGTCATCATTCCTGAGCAAGTGCTGGACTCAGATGTAACTGAAGAAGTTTCTTTAGCACATTGCACAGTCCCAGATGATGTTTTAGCTTCTGACATTACTTCAGCCTCAATGTCTATGCCAGAACACGTCTTGACGGGTGATTCTATACATGTGTCTGACGTTGGACATGTTGGACATGTTGGACATGTTGAACATGTGGTTCATGATAGTGTAGTGGAAGCAGAAATTGTCACTGATCCTCTGACTACCGACGTAGTTTCAGAAGAAGTATTGGTAGCAGACTGTGCCTCTGAAGCAGTCATAGATGCCAATGGGATCCCTGTGGACCAGCAGGATGATGACAAAGGCAACTGTGAGGACTACCTTATGATTTCCTTGGATGATGCTGGCAAAATAGAACACGATGGTTCTTCTGGAATGACCATGGACACAGA**GTCGGAAATTGATCCTTGTAA**AGTGGATGGCACTTGCCCTGAGGTCATCAAGGTGTACATTTTTAAAGCTGACCCTGGAGAAGATGACTTAGGTGGAACTGTAGACATTGTGGAGAGTGAGCCTGAGAATGATCATGGAGTTGAACTGCTTGATCAGAACAGCAGTATTCGTGTTCCCAGGGAAAAGATGGTTTATATGACTGTCAATGACTCTCAGCCAGAAGATGAAGATTTAAATGTTGCTGAAATCGCTGACGAAGTTTATATGGAAGTGATCGTAGGAGAGGAGGATGCTGCAGCAGCAGCGGCAGCCGCCGCCGTGCACGAGCAGCAAATGGATGACAATGAAATCAAAACCTTCATGCCGATTGCATGGGCAGCAGCTTATGGTAATAATTCTGATGGAATTGAAAACCGGAATGGCACTGCAAGTGCCCTCTTGCACATAGATGAGTCTGCTGGCCTCGGCAGACTGGCTAAACAAAAACCAAAGAAAAGGAGAAGACCTGATTCCAGGCAGTACCAAACAGCAATAATTATTGGCCCTGATGGACATCCTTTGACTGTCTATCCTTGCATGATTTGTGGGAAGAAGTTTAAGTCGAGAGGTTTTTTGAAAAGGCACATGAAAAACCATCCCGAACACCTTGCCAAGAAGAAATACCGCTGTACTGACTGTGATTACACTACCAACAAGAAGATAAGTTTACACAACCACCTGGAGAGCCACAAGCTGACCAGCAAGGCAGAGAAGGCCATTGAATGCGATGAGTGTGGGAAGCATTTCTCTCATGCAGGGGCTTTGTTTACTCACAAAATGGTGCATAAGGAAAAAGGAGCCAACAAAATGCACAAGTGTAAATTCTGTGAATACGAGACAGCTGAACAAGGGTTATTGAATCGCCACCTCTTGGCAGTCCACAGCAAGAACTTTCCTCATATTTGTGTGGAGTGTGGTAAGGGTTTTCGTCACCCGTCAGAGCTCAAAAAGCACATGAGAATCCATACTGGGGAGAAGCCGTACCAATGCCAGTACTGCGAATATAGGTCTGCAGACTCTTCTAACTTGAAAACGCATGTCAAAACTAAGCATAGTAAAGAGATGCCATTCAAGTGTGACATTTGTCTTCTGACTTTCTCGGATACCAAAGAGGTGCAGCAACATGCTCTTATCCACCAAGAAAGCAAAACACACCAGTGTTTGCATTGCGACCACAAGAGTTCGAACTCAAGTGATTTGAAACGACACATAATTTCAGTTCACACGAAAGACTACCCCCATAAGTGTGACATGTGTGATAAAGGCTTTCACAGGCCTTCAGAACTCAAGAAACACGTGGCTGCCCACAAGGGCAAAAAAATGCACCAGTGTAGACATTGTGACTTTAAGATTGCAGATCCATTTGTTCTAAGTCGCCATATTCTCTCAGTTCACACAAAGGATCTTCCATTTAGGTGCAAGAGATGTAGAAAGGGATTTAGGCAACAGAGTGAGCTTAAAAAGCATATGAAGACACACAGTGGCAGGAAAGTGTATCAGTGTGAGTACTGTGAGTATAGCACTACAGATGCCTCAGGCTTTAAACGGCACGTTATTTCCATTCACACGAAAGACTATCCTCACCGGTGTGAGTACTGCAAGAAAGGCTTCCGAAGACCTTCAGAAAAGAACCAGCACATAATGCGACATCATAAAGAAGTTGGCCTGCCCTAA |
| Mutant-type ZFX | |
|  | *>gi\|296010876:308-2725 Homo sapiens zinc finger protein, X-linked (ZFX), transcript variant 1, mRNA*  ATGGATGAAGATGGGCTTGAATTACAACAAGAGCCAAACTCATTTTTTGATGCAACAGGAGCTGATGGTA  CACACATGGATGGTGATCAAATTGTTGTGGAAGTACAAGAAACTGTTTTTGTTTCAGATGTTGTGGATTC  AGACATAACTGTGCATAACTTTGTTCCTGATGACCCAGATTCAGTTGTAATCCAAGATGTTATTGAGGAC  GTTGTTATAGAAGATGTTCAGTGCCCAGATATCATGGAAGAAGCAGATGTGTCTGAAACGGTCATCATTC  CTGAGCAAGTGCTGGACTCAGATGTAACTGAAGAAGTTTCTTTAGCACATTGCACAGTCCCAGATGATGT  TTTAGCTTCTGACATTACTTCAGCCTCAATGTCTATGCCAGAACACGTCTTGACGGGTGATTCTATACAT  GTGTCTGACGTTGGACATGTTGGACATGTTGGACATGTTGAACATGTGGTTCATGATAGTGTAGTGGAAG  CAGAAATTGTCACTGATCCTCTGACTACCGACGTAGTTTCAGAAGAAGTATTGGTAGCAGACTGTGCCTC  TGAAGCAGTCATAGATGCCAATGGGATCCCTGTGGACCAGCAGGATGATGACAAAGGCAACTGTGAGGAC  TACCTTATGATTTCCTTGGATGATGCTGGCAAAATAGAACACGATGGTTCTTCTGGAATGACCATGGACA  CAGA**GAGCGAGATCGACCCCTGCAA**AGTGGATGGCACTTGCCCTGAGGTCATCAAGGTGTACATTTTTAA  AGCTGACCCTGGAGAAGATGACTTAGGTGGAACTGTAGACATTGTGGAGAGTGAGCCTGAGAATGATCAT  GGAGTTGAACTGCTTGATCAGAACAGCAGTATTCGTGTTCCCAGGGAAAAGATGGTTTATATGACTGTCA  ATGACTCTCAGCCAGAAGATGAAGATTTAAATGTTGCTGAAATCGCTGACGAAGTTTATATGGAAGTGAT  CGTAGGAGAGGAGGATGCTGCAGCAGCAGCGGCAGCCGCCGCCGTGCACGAGCAGCAAATGGATGACAAT  GAAATCAAAACCTTCATGCCGATTGCATGGGCAGCAGCTTATGGTAATAATTCTGATGGAATTGAAAACC  GGAATGGCACTGCAAGTGCCCTCTTGCACATAGATGAGTCTGCTGGCCTCGGCAGACTGGCTAAACAAAA  ACCAAAGAAAAGGAGAAGACCTGATTCCAGGCAGTACCAAACAGCAATAATTATTGGCCCTGATGGACAT  CCTTTGACTGTCTATCCTTGCATGATTTGTGGGAAGAAGTTTAAGTCGAGAGGTTTTTTGAAAAGGCACA  TGAAAAACCATCCCGAACACCTTGCCAAGAAGAAATACCGCTGTACTGACTGTGATTACACTACCAACAA  GAAGATAAGTTTACACAACCACCTGGAGAGCCACAAGCTGACCAGCAAGGCAGAGAAGGCCATTGAATGC  GATGAGTGTGGGAAGCATTTCTCTCATGCAGGGGCTTTGTTTACTCACAAAATGGTGCATAAGGAAAAAG  GAGCCAACAAAATGCACAAGTGTAAATTCTGTGAATACGAGACAGCTGAACAAGGGTTATTGAATCGCCA  CCTCTTGGCAGTCCACAGCAAGAACTTTCCTCATATTTGTGTGGAGTGTGGTAAGGGTTTTCGTCACCCG  TCAGAGCTCAAAAAGCACATGAGAATCCATACTGGGGAGAAGCCGTACCAATGCCAGTACTGCGAATATA  GGTCTGCAGACTCTTCTAACTTGAAAACGCATGTCAAAACTAAGCATAGTAAAGAGATGCCATTCAAGTG  TGACATTTGTCTTCTGACTTTCTCGGATACCAAAGAGGTGCAGCAACATGCTCTTATCCACCAAGAAAGC  AAAACACACCAGTGTTTGCATTGCGACCACAAGAGTTCGAACTCAAGTGATTTGAAACGACACATAATTT  CAGTTCACACGAAAGACTACCCCCATAAGTGTGACATGTGTGATAAAGGCTTTCACAGGCCTTCAGAACT  CAAGAAACACGTGGCTGCCCACAAGGGCAAAAAAATGCACCAGTGTAGACATTGTGACTTTAAGATTGCA  GATCCATTTGTTCTAAGTCGCCATATTCTCTCAGTTCACACAAAGGATCTTCCATTTAGGTGCAAGAGAT  GTAGAAAGGGATTTAGGCAACAGAGTGAGCTTAAAAAGCATATGAAGACACACAGTGGCAGGAAAGTGTA  TCAGTGTGAGTACTGTGAGTATAGCACTACAGATGCCTCAGGCTTTAAACGGCACGTTATTTCCATTCAC  ACGAAAGACTATCCTCACCGGTGTGAGTACTGCAAGAAAGGCTTCCGAAGACCTTCAGAAAAGAACCAGC  ACATAATGCGACATCATAAAGAAGTTGGCCTGCCCTAA |

| **Supplementary Table 5. Sequence of siRNAs Targeting β-catenin in This Study** | | |
| --- | --- | --- |
|  | Sense | Antisense |
| siβ-Catenin 1# | GAUGGUGUCUGCUAUUGUACG | UACAAUAGCAGACACCAUCUG |
| siβ-Catenin 2# | GGACAAGGAAGCUGCAGAAGC | UUCUGCAGCUUCCUUGUCCUG |
| siβ-Catenin 3# | GAAUACAAAUGAUGUAGAAAC | UUCUACAUCAUUUGUAUUCUG |

| **Supplementary Table 6. Sequence of PCR Primers Used in This Study** | | |
| --- | --- | --- |
| Gene | Forward primer | Reverse primer |
| ZFX | 5'-GGCAGTCCACAGCAAGAAC-3' | 5'-TTGGTATCCGAGAAAGTCAGAAG-3' |
| CD90 | 5'-CTAGTGGACCAGAGCCTTCG-3' | 5'-GCACGTGCTTCTTTGTCTCA-3' |
| CD133 | 5'-GCCACCGCTCTAGATACTGC-3' | 5'-TGTTGTGATGGGCTTGTCAT-3' |
| Sox2 | 5'-CAAGATGCACAACTCGGAGA-3' | 5'-GCTTAGCCTCGTCGATGAAC-3' |
| Oct4 | 5'-AGTGAGAGGCAACCTGGAGA-3' | 5'-ACACTCGGACCACATCCTTC-3' |
| Nanog | 5'-CTGCTGGACTGAGCTGGTTGCC-3' | 5'-GCTGAGGCCTTCTGCGTCACA-3' |
| Bmil | 5'-AGAGATCGGGGCGAGACAAT-3' | 5'-TTGCTGGTCTCCAGGTAACG-3' |
| Notch-1 | 5'-TCCACCAGTTTGAATGGTCA-3' | 5'-CGCAGAGGGTTGTATTGGTT-3' |
| EpCAM | 5'-CTGCCAAATGTTTGGTGATG-3' | 5'-AAAGCCCATCATTGTTCTGG-3' |
| Actin | 5'-CCCTGGCACCCAGCAC-3' | 5'-GCCGATCCACACGGAG-3' |
| c-Myc | 5'-AGGACGCGACTCTCCCGACG-3' | 5'-CGCAACGTAGGAGGGCGAGC-3' |
| Cyclin D1 | 5′-CTGCTGCGAAGTGGAAACCAT-3′ | 5′-TTCATGGCCAGCGGGAAGACCTC-3′ |
| c-Jun | 5′-CCTATCCCCTGTGTGCCTTGGC-3′ | 5′-CAGTCTCAGAGAATTATGGCGT-3′ |

**Part 3. Supplementary Figure**

Supplementary Figure 1 (Related to Figure 4).

**
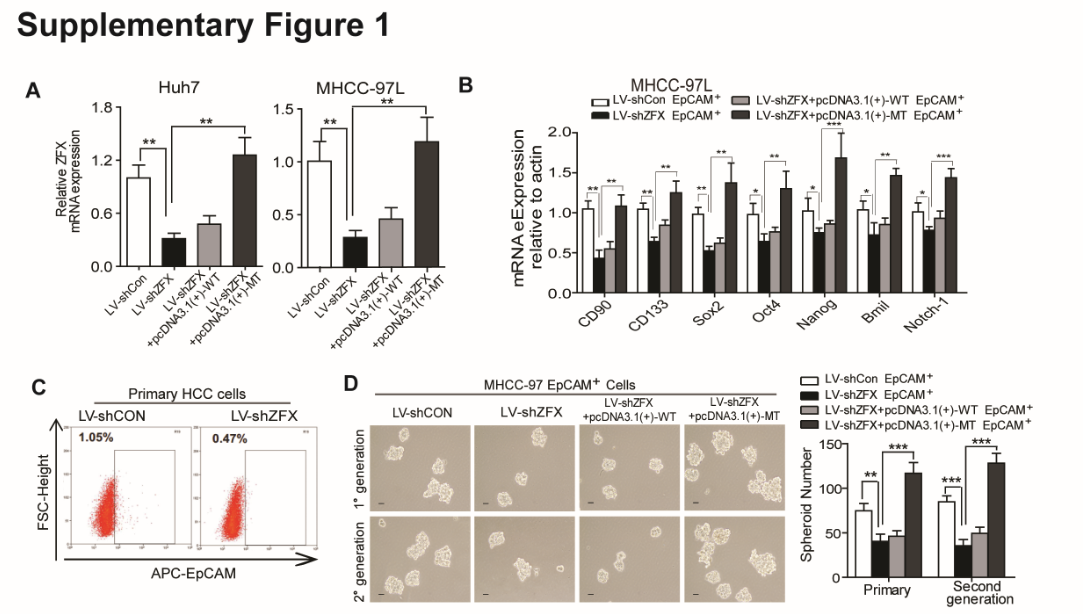
**

**
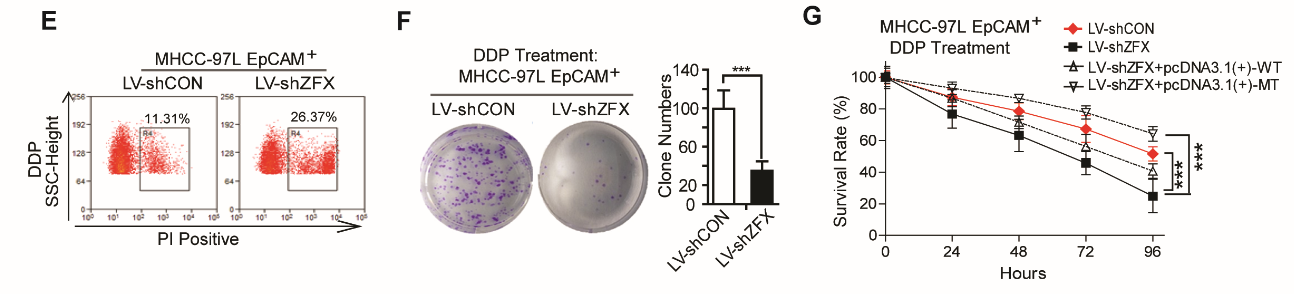
**

**ZFX is required to maintain stem cell-like features of EpCAM^+^ liver CSCs.**

MHCC-97L and Huh7 cells were firstly infected with LV-shCon and LV-shZFX, then the HCC cells stably expressing shZFX were transfected with wide-type (WT) or mutant-type (MT) ZFX as mentioned in Method section. (A) The mRNA expression levels of ZFX in MHCC-97L and Huh7 cells with ZFX depletion or re-expression were detected by qRT-PCR. (B) EpCAM^+^ cancer cells were magnetically sorted from ZFX-silenced or re-expressed MHCC-97L cells, and their mRNA expression levels of multiple stemness-related genes were evaluated by qRT-PCR. In (A) and (B), the fold change was determined using the delta-delta Ct method. Quantified mRNA levels were normalized to β-actin and presented relative to the controls. Representative results from three independent experiments are shown and all data represent Mean ± SD. *p<0.05, **p<0.01 and ***p<0.001. (C) Freshly isolated primary HCC cells were infected with LV-shCon or LV-shZFX for 48 hours. Then the percentage of EpCAM^+^ subpopulation in HCC cells stably expressing shCon or shZFX was measured via flow cytometric assay. (D) Representative images of primary and secondary passaged HCC spheriods derived from EpCAM^+^ MHCC-97L cells with ZFX knockdown or re-expression are shown (Scale bar = 100 μm) (left pannel), and the number of spheriods was counted. Experiments were performed in triplicate and all data are shown as Mean ± SD. **p<0.01 and ***p<0.001. (E) MHCC-97L LV-shCon EpCAM^+^ or LV-shZFX EpCAM^+^ cells were treated with 2 μg/mL [cisplatin](http://cn.bing.com/dict/search?q=cisplatin&FORM=BDVSP6&mkt=zh-cn) (DDP) for 4 days and PI staining was performed to detect the proportion of dead cells via flow cytometry. (F) The colony formation capacity of MHCC-97L LV-shCon EpCAM^+^ or LV-shZFX EpCAM^+^ cells in the presence of 1 μg/mL DDP for 2 weeks. Representative images of colony formation are shown (left panel) and colony number was counted. (G) The indicated HCC cells were treated with DDP (2 μg/mL) and cell viability was detected by CCK-8 assay. In (E), (F) and (G), representative results from three independent experiments are shown and all data represent Mean ± SD. ***p<0.001.

**Supplementary Figure 2 (Related to Figure 4).**

**
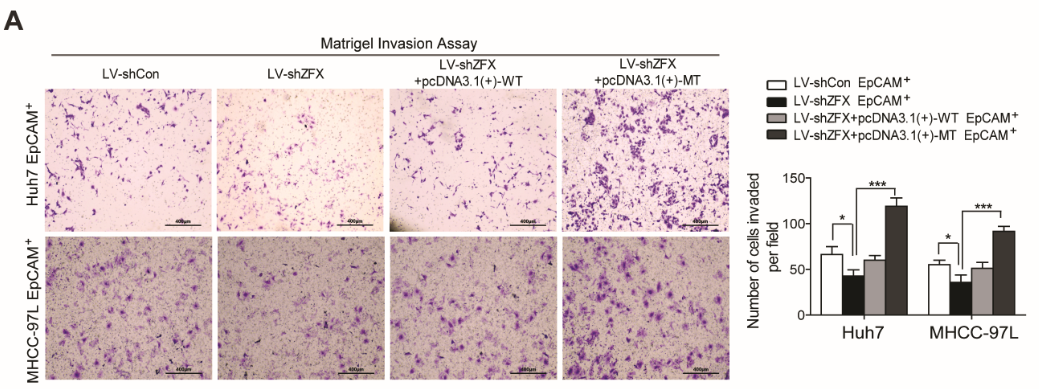
**

**
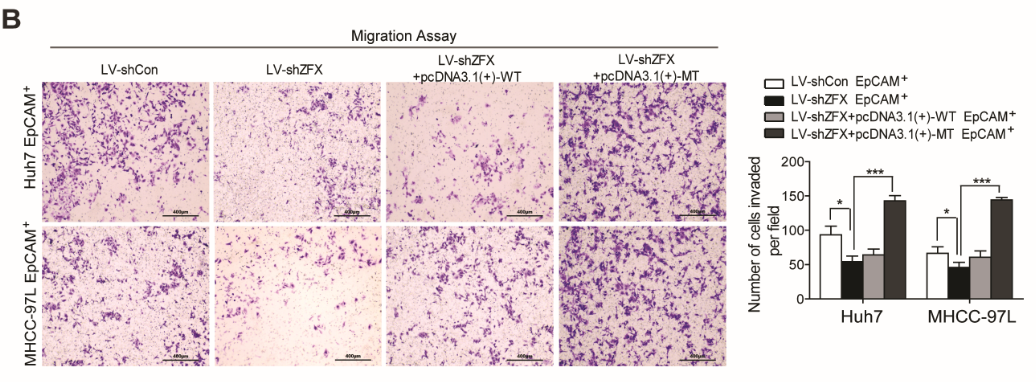
**

**The impact of ZFX on EpCAM^+^ HCC cell invasion and migration *in vitro*.**

(A) The invasive properties of EpCAM^+^ HCC cells expressing shZFX or ZFX plasmid were analyzed with the invasion assay using BioCoat Matrigel invasion as described above. (B) The migratory properties of the cells were analyzed using the Transwell migration assay with Transwell filter chambers. The average numbers of invasive and migrated cells from 5 random microscopic fields are calculated and are presented as Mean ± SD. *P < 0.05, **p<0.01 and ***p<0.001 compared with cells infected with LV-shCon. Scale bar = 400 μm. Magnification: 100x.

**Supplementary Figure 3 (Related to Figure 6).**


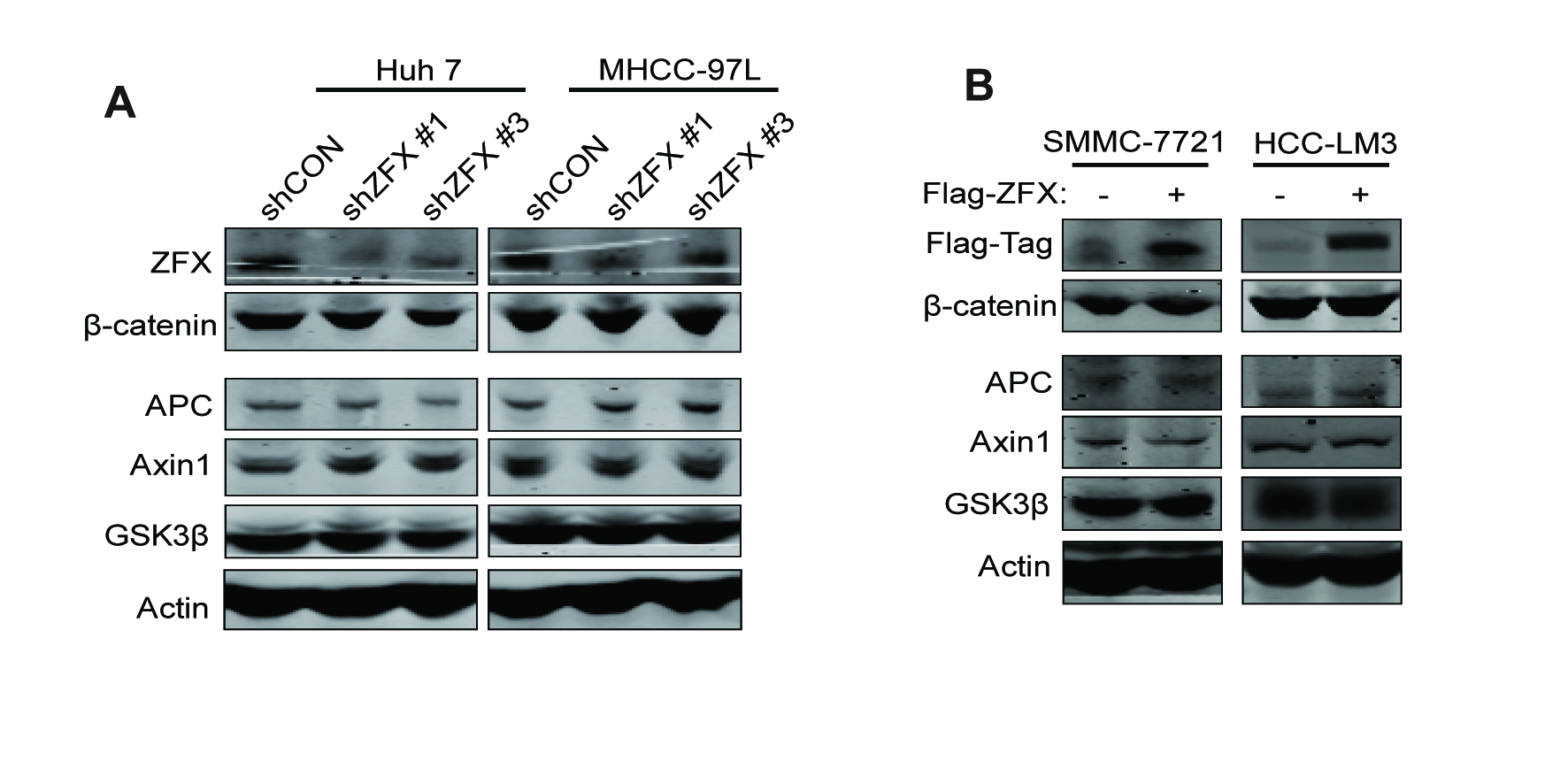


(A) The expression levels of ZFX, β-catenin, APC, Axin1, and GSK3β in Huh7 and MHCC-97L cells stably expressing shCon or shZFX (sequence #1 and #3) were detected by western blot assays. (B) The protein contents of indicated molecules in SMMC-7721 and HCCLM3 cells transfected with empty vector or Flag-tagged ZFX plasmids were determined by western blot assays. β-actin was used as internal loading control and representative results from three independent experiments are shown.
